# The global distribution of bamboos: assessing correlates of introduction and invasion

**DOI:** 10.1093/aobpla/plw078

**Published:** 2016-12-23

**Authors:** Susan Canavan, David M. Richardson, Vernon Visser, Johannes J. Le Roux, Maria S. Vorontsova, John R. U. Wilson

**Affiliations:** 1 Centre for Invasion Biology, Department of Botany and Zoology, Stellenbosch University, Matieland 7602, South Africa; 2 Invasive Species Programme, South African National Biodiversity Institute, Kirstenbosch Research Centre, Private Bag X7, Claremont 7735, South Africa; 3 SEEC—Statistics in Ecology, Environment and Conservation, Department of Statistical Sciences, University of Cape Town, Rondebosch 7701, South Africa; 4 African Climate and Development Initiative, University of Cape Town, Cape Town, Rondebosch 7701, South Africa; 5 Comparative Plant & Fungal Biology, Royal Botanic Gardens, Kew, Richmond, Surrey TW9 2AB, UK

**Keywords:** Bamboo, Bambusoideae, biological invasions, cultivars, introduced species, invasive species, Poaceae

## Abstract

There is a long history of species being moved around the world by humans. These introduced species can provide substantial benefits, but they can also have undesirable consequences. We explore the importance of human activities on the processes of species dissemination and potential invasions using the Poaceae subfamily Bambusoideae (‘bamboos’), a group that contains taxa that are widely utilised and that are often perceived as weedy. We (1) compiled an inventory of bamboo species and their current distributions; (2) determined which species have been introduced and become invasive outside their native ranges; and (3) explored correlates of introduction and invasion. Distribution data were collated from Kew’s GrassBase, the Global Biodiversity Information Facility and other online herbarium information sources. Our list comprised 1662 species in 121 genera, of which 232 (14 %) have been introduced beyond their native ranges. Twelve (0.7 % of species) were found to be invasive. A non-random selection of bamboos have been introduced and become invasive. Asiatic species in particular have been widely introduced. There was a clear over-representation of introduced species in the genera *Bambusa* and *Phyllostachys* which also contain most of the listed invasive species. The introduction of species also correlated with certain traits: taxa with larger culm dimensions were significantly more likely to have been moved to new areas; and those with many cultivars had a higher rate of dissemination and invasion. It is difficult to determine whether the patterns of introduction and invasion are due simply to differences in propagule pressure, or whether humans have deliberately selected inherently invasive taxa. In general, we suggest that human usage is a stronger driver of introductions and invasions in bamboos than in other taxa that have been well studied. It is likely that as bamboos are used more widely, the number and impact of invasions will increase unless environmental risks are carefully managed.

## Teaser

Bamboos are one of the most economically important plant groups globally, but this world-wide trade creates risks of invasions. Here we identified 1662 species of bamboo, of which almost a seventh (232) have been introduced outside their native range, with Asiatic and larger species preferred. Only 12 species were found to have become invasive. However, invasiveness was found to be more a function of human-usage than specific species traits, suggesting the recent upsurge in bamboo cultivation could pose future invasion risks.

## Introduction

Human-mediated dissemination of species has intensified over the past three centuries with the increase of global traffic (Meyerson and Mooney 2007; Ricciardi 2007). Some introduced species naturalize (reproduce consistently) in their new ranges and some naturalized species invade (spread from sites of introduction). This has created a global-scale natural experiment in biogeography (Bardsley and Edward-Jones 2006; Richardson 2006; Richardson *et al*. 2011a; Richardson *et al*. 2011c; Yoshida *et al*. 2007). Considerable efforts have been made by invasion scientists to understand the key drivers of invasion, and to determine whether generalisations can be made on how some species manage to overcome barriers associated with different stages of the introduction-naturalization-invasion continuum (Blackburn *et al.* 2011; Kueffer *et al.* 2013; Moodley *et al.* 2016; Richardson and Pyšek 2012). However, as introduced taxa often represents a non-random selection of all taxa, there is some ‘taxonomic selectivity’ in which taxa become invasive (McKinney and Lockwood 1999).

Biological invasions are, by definition, the result of human-mediated dispersal and can only be understood in the context of human activities. The movement of species is often influenced by their direct value to humans (McKinney and Lockwood 1999), in particular as introduced species have been essential to the development of all contemporary human societies (Prance and Nesbitt 2005). With intentional plant introductions, morphological traits have been shown to be important in facilitating the introduction and invasion of species (Pyšek and Richardson 2007). Certain traits may be of high value to humans at the introduction stage and thus influence the initial movement of these species into new ranges. For example, Proteaceae with showy flowers and Cactaceae with other traits valued for ornamentation were found to be overrepresented among introduced species in these families (Moodley *et al.* 2013; Novoa *et al.* 2015). For both these families, traits that enabled greater ability to spread were found to be more important for invasion success post-introduction. Traits underlying invasion success can also be highly taxon or context specific. In many woody plant taxa, such as *Acacia*, *Pinus* and Proteaceae, seedbank size and longevity are associated with invasion success (Grotkopp *et al.* 2002; Moodley *et al.* 2013; Richardson and Kluge 2008), while in Cactaceae growth form is an important determinant of invasion success. (Novoa *et al.* 2015).

We focused on bamboos, a large subfamily of the grasses (Poaceae: Bambusoideae; 1662 species in 121 genera). Bamboos have a range of functional forms distributed over numerous biogeographic regions, including dwarf herbaceous species found in temperate climates and giant tropical woody species that can grow up to 20 m tall (Bystriakova *et al.* 2004). It is estimated that 2.5 billion people are directly involved with the production and consumption of bamboo (Scurlock *et al.* 2000). The main economic value of bamboo lies in the utility of the hardened culm, which serves many of the same functions as timber (Chung and Yu 2002; Scurlock *et al.* 2000). What makes bamboo a particularly interesting group beyond timber functions, however, is the versatility of uses and the utilisation of all plant parts. Leaves are used for fodder, shoots for human consumption, culms for biomass, construction, textiles, musical instruments and many bamboos are used in horticulture (Hunter 2003). This has led to many species being intentionally moved outside of their native ranges (Cook and Dias 2006; Townsend 2013).

Over the past few decades, bamboos have seen an upsurge in popularity, largely driven by a perception of certain species as wonder plants or miracle crops, i.e. plants that are believed to be especially valuable in meeting current economic, environmental and social needs (Hoogendoorn and Benton 2014; Liese and Köhl 2015). Various authors have argued that commercially grown bamboos are more sustainable and renewable than current forestry crops (Bansal and Zoolagud 2002; Song *et al.* 2011). Modern processing techniques have also transformed the range of products that can be made from bamboo. Therefore, the rate at which species are being introduced and cultivated in new ranges has increased; especially cultivation of bamboos in response to an increased global demand for timber products (Hunter 2003; INBAR 2003).

Most research on bamboos has focused on aspects of commercial cultivation and uses such as methods for maximizing yields and on providing economic valuations of plantings in different contexts. To date, we are not aware of any comprehensive studies on the invasion ecology of bamboos, despite their reputation for being a group that contains highly ‘invasive’ species (Buckingham *et al.* 2011; Space and Flynn 2000). Many species possess weedy attributes, such as fast growth rates, clonal reproduction and the formation of long-lived monospecific stands (Lima *et al.* 2012). Bamboos can dramatically alter ecosystem dynamics through competitive exclusion and expansion of patches that form from clonal reproduction**.** A growing number of papers address some of these issues (Blundell *et al.* 2003; Kobayashi *et al.* 2015; Kudo *et al.* 2011; Lima *et al.* 2012; Rother *et al.* 2016; Suzuki 2015; Yang *et al.* 2015).

While there has been a long history of bamboo introductions, little is known about which species have been moved where, and the outcomes of these movements. The aims of this paper were to (1) compile an inventory of all bamboo species and their current global distribution; (2) determine which species have been introduced and which have become invasive outside of their native ranges; and (3) explore correlates of introduction and invasion. We expected that certain correlates, both biological (i.e. taxonomy, phylogeny, plant traits) and social (i.e. introduction effort, the utility of species), will have resulted in taxonomic selectivity in introduction effort (Table 1).

**Table 1:** Features correlated with the introduction and invasion status of bamboos.

Correlate/measurement	Expectation	Result	Consequence	Figure/table in this paper
Taxonomy (genera)	Introduced species will tend to come from certain genera	The genera *Bambusa*, *Phyllostachys*, *Semiarundinaria*, *Shibataea*, and *Thyrsostachys* had a significant proportion of species that have been introduced; and *Bambusa*, *Phyllostachys* and *Pleioblastus* had a significant proportion of species that were invasive (both relative to other genera)	The pool of introduced species is a very particular subset of all bamboos, so need to be careful about assessing traits linked to invasiveness only on introduced taxa	Fig. 4
Phylogeny	There will be a non-random assortment of which species are introduced across the phylogeny	Only culm height showed significant phylogenetic signal, other variables including status were not		See Fig. S2
Lineage (neotropical woody, etc.)	Taxa from particular biogeographical regions are more likely to become introduced (even if phylogeny and introduction history are taken into account)	Temperate bamboos have had a high rate of species introduced compared with other lineages. Both temperate and paleotropical woody bamboos contain invasive species, but neither had a significant number compared with the other	Bamboos from other parts of the world are likely to have significant potential for utilisation in the future. Region of origin could be an important correlate of risk	Table 2
Number of countries/regions a species have been introduced to	Species of bamboo that have been introduced to many ranges will have a higher likelihood of becoming invasive	The number of countries a species has been introduced to was strongly (positively) correlated with the likelihood of it being invasive	Risk and impacts caused by non-native bamboos are a function of propagule pressure	See text for details
Number of cultivars	Species with a greater number of cultivars will be more likely to have been introduced than species with fewer cultivars	Introduced species tended to have more cultivars	There has been a possible selection for species that show high levels of phenotypic variation, this can potentially be linked to a greater ability to adapt and so become invasive. On the other hand, more efforts may have simply been made to develop cultivars for common species	See text for details
	Species with many cultivars will have a higher likelihood of becoming invasive	Greater number of cultivars was an important determinant of invasion	Invasiveness has been selected for during breeding and cultivation practices	
Culm form	Woody lineages will have a higher proportion of introduced species than herbaceous.	Woody bamboos are preferred for introduction	As herbaceous species have had much lower rates of introduction, there has been a bias in the natural experiment.	Table 2
Culm dimensions (diameter and height)	Introduced species will on average have greater culm dimensions than non-introduced species	There is an affinity for species to be introduced that have greater culm dimensions	Smaller bamboos will be less likely to have been introduced.	Fig. 5
Rhizome form (running or clumping species)	Introduced bamboo species with running rhizomes are more likely to become invasive, although there is no prior expectation as to how this might affect which species are introduced	Rhizome form was not an indicator of invasive species. However, we did find more running type bamboos have been introduced (although this is correlated with temperate species which have had a bias for introduction)	Control and regulation of bamboos should consider both running and clumping forms	Table 2

## Methods

### Inventory of species and distribution

Establishing inventories of taxa, their distribution and cases of invasions are fundamentally important in the field of invasion science and the lack of such information can hinder management efforts (McGeoch *et al.* 2012). To document the dissemination of bamboos, we required up-to-date taxonomic lists and distribution data.

The identification of bamboos is notoriously problematic (reviewed by Kellogg 2015). Due to the rarity of flowering cycles (7 to more than 120 years in woody species; Janzen 1976), species identification often relies heavily on vegetative material, but most species have few, if any, reliable diagnostic vegetative features. Consequently, there are major discrepancies between the classification of bamboos and species lists. Significant improvements have been made by specialist groups such as the Bamboo Phylogeny Group (2012) and, more generally, by GrassBase, an on-going international initiative to collate taxonomic data on the family Poaceae at the Royal Botanical Gardens, Kew, UK. GrassBase includes a list of all bamboo species, their distributions and trait data (Clayton *et al.* 2015; Vorontsova *et al.* 2015). We verified and updated the accepted taxa in GrassBase both as one of us has specialist experience in grass taxonomy (MSV) and by collaborating with a bamboo taxonomy specialist (Lynn G. Clark, Iowa State University). We also included recent literature on new species and other changes in classification published up to September 2015 (Kellogg 2015) **[see Supporting Information—Table S1 for full species list]**.

An extensive search was undertaken between June 2014 and January 2015 to document the introduction of bamboos to areas outside of their native ranges. This included searches of the Web of Science and other platforms of academic and grey literature. Most information was retrieved from online databases specialising in global herbarium records and/or non-native species records, namely the Global Biodiversity Information Facility (GBIF), Kew’s GrassBase, the Global Compendium of Weeds (GCW), Pacific Island Ecosystems at Risk (PIER), Delivering Alien Invasive Species Inventories for Europe (DAISIE), Invasive Species Specialist Group (ISSG) and CABI’s Invasive Species Compendium (CABI-ISC), but independent literature searches also provided useful data **[see Supporting Information—Fig. S1].** GBIF provided the greatest amount of data on the locality of species with over 84 000 entries for ‘Bambusoideae’ species. Of these, around 29 % of records had sufficient ancillary data for our purposes (of the 71% that did not, 8 % lacked a scientific name, 21 % a country and 71 % a locality)

When pooled with the other databases, 179 species names did not match our accepted species list. Unknown names were removed; synonyms and spelling errors were updated or corrected accordingly and kept in the final database **[see Supporting Information—Table S2]**. We discarded records on the basis of names that we could not resolve using these criteria. The final list for analyses included over 27 000 entries. Names of geographic regions were defined based on the International Organization for Standardization for country codes and regions (ISO 31661-1 standard; with the exception of a few island regions which were independently defined, such as Hawaii and the Galapagos Islands).

### Dissemination and status

We categorized the presence of a species in a given country or region as native or non-native (or introduced) based on distribution data from Kew’s GrassBase and cross-referenced with Ohrnberger (1999). These two data sources provide a complete inventory of the taxonomy and distribution of bamboos that was needed to establish native and introduced ranges. We defined these categories using the compendium of concepts in invasion science proposed by Richardson *et al.* (2011). Species were listed as ‘non-native’ or ‘introduced’ when their presence in a region is due to human activity. Note that our records do not distinguish between successful introductions (where species have established and are still present today) and failed introductions (where species no longer occur in that region)—they simply reflect the presence of a species in a given region at some point in time. We classified a subset of ‘non-native’ species as ‘invasive’. Invasive species are ‘naturalized plants that produce reproductive offspring often in large numbers at a considerable distance from parent plants…’ (Richardson *et al*. 2011b). Records of bamboos being listed as invasive were found either through the databases mentioned above, or through an independent literature search. References for invasions came from a combination of peer-reviewed literature and official government reports, which were then cross-checked to validate claims that species were ‘invasive’ following the criteria of Richardson *et al.* (2011b)**[see Supporting Information—Table S3].**

To conceptualize and display the flows of introduced and invasive species between and within different biogeographic regions around the world, we used circos visualization from the R package ‘circlize’ (Gu *et al*. 2014).

### Correlates of introduction and invasion

*Morphological traits*: To determine whether particular traits were related with the introduction status and invasion success of bamboos, we collated trait data from GrassBase. The dataset included 14 trait categories (culms, culm-sheaths, leaves, ligule, etc.). However, only culm dimensions (diameter and height) and underground rhizome system (runner or clumper) were consistently recorded (data on other traits were not available for more than half of the species). These traits were chosen as they were considered relevant to the study and data were available for many of the species.

Different culm properties provide different benefits—thicker-walled culms yield more biomass, greater diameter can produce stronger culms, etc. (Chung and Yu 2002; Scurlock *et al.* 2000). To determine whether introduced and/or invasive species had taller and/or wider culms than non-introduced species, we used linear models with log-transformed culm dimension (height or diameter) as a response variable and introduction status as the predictor variable. We also included lineage affiliation (paleotropical woody, neotropical woody, temperate woody and herbaceous) as an additional predictor as these have been identified as genetically distinct groups within bamboos that have particular growth forms associated with each (Kelchner *et al.* 2013). We also tested the differences in culm form of woody versus herbaceous groups in a number of introduced species compared with non-introduced species, and the number of invasive compared with non-invasive species using Fisher’s exact tests. All statistical tests were conducted in R (R Core Team 2015).

Underground rhizome type was also considered a relevant trait for invasion success, as it is often used as a means of separating invasive from non-invasive bamboos (Hamilton 2010; Royal Horticultural Society 2015). There are two forms: running (leptomorph) and clumping (pachymorph). Although sub-forms exist within these categories, for simplicity we only used these two broad categories. Running species are considered to have a greater ability to spread rapidly and are generally considered more invasive than clumping species (Buckingham *et al* 2014). To test the difference in number of running and clumping species in the groups of introduced compared with non-introduced, and the number of invasive compared with non-invasive species, we used Fisher’s exact tests.

#### Taxonomic, geographic and phylogenetic patterns

The exchange of species and the rates of invasion are rarely random, but often have distinct patterns that are influenced by a number of factors, some human-mediated and others related to the evolutionary history of species. Within particular groups this can lead to ′taxonomic selectivity′. In the case of bamboo, forestry and horticulture have been the main drivers of introductions, and this has led to the preferential selection of taxa. To test whether introductions and invasions have been random, we used Fisher’s exact test to analyse differences between numbers of introduced compared with non-introduced species, and the number of invasive compared with non-invasive species across genera, lineages (i.e. neotropical woody), and introduced countries.

If certain bamboo traits are important to invasion success, and if these traits reflect evolutionary history, then we would expect the phylogeny to indicate ′taxonomic selectivity′, with only certain lineages becoming invasive. Much work has been done on reviewing this phenomenon to improve the prediction of extinctions. Studies have found that extinctions within taxonomic groups in birds, mammals and plants tend not to be randomly distributed across phylogenies but are concentrated in particular high-risk clades (Fritz and Purvis 2010; McKinney and Lockwood 1999). This is arguably due to phylogenetically conserved life-history traits or ecology (Fritz and Purvis 2010; Purvis 2008; Schwartz and Simberloff 2001; Thomas 2008). There is evidence to suggest this is also true with invasiveness across taxa (Lockwood 1999; Lockwood *et al.* 2001; Lockwood and McKinney 2001; Novoa *et al.* 2015; Yessoufou *et al.* 2016). We explore this for bamboos by testing the phylogenetic signal of status (introduced/invasive) and other correlates of introduction and invasion. To do this we collated genetic data for one chloroplast gene region (maturase K; *matK*) for all taxa with available data in the online GenBank repository (ncbi.nlm.nih.gov) for phylogeny reconstruction. Where possible, GenBank accessions denoted as ‘voucher’ specimens were used. Our final dataset comprised 124 taxa (including two non-bamboo grass species *Bromus interruptus* & *Trisetum spicatum* as outgroup taxa). DNA sequence data were combined and aligned in the BioEdit version 7.0.5.3 (Hall 2006) and were edited manually. Flanking regions were trimmed to avoid excessive missing data. Our final DNA alignment consisted of 860 characters and contained three gaps ranging between 1 and 6 base pairs. A Bayesian inference phylogeny was reconstructed using Mr Bayes v 3.2 (Ronquist and Huelsenbeck 2003). jModelTestv2.13 (Darriba *et al.*, 2012) and the Akaike information criterion (Akaike, 1973) determined the best fit model for our data as the GTR + I +G model. The Bayesian model was run for 1.5 million generations sampling every 1000th generation and a consensus tree was built, discarding the first 25 % of trees as burn-in. Posterior probabilities (PP) were calculated using a majority rule consensus method to assess tree topology support.

To test whether continuous traits (culm dimensions) are phylogenetically clustered or over-dispersed, we used Blomberg’s K statistic with a null hypothesis of Brownian Motion Model (Blomberg *et al.* 2003). We also tested for phylogenetic signal of other variables, i.e. introduction and invasion frequency (the number of countries a species has been introduced to or become invasive), and propagule pressure (using the frequency of cultivars as a proxy; see below) using Pagel’s *λ* (lambda) which uses transformation of the branch lengths assuming Brownian motion (Pagel 1999). Both analyses were done using the R packages ‘phytools’ and function Phylosig.R (Revell, 2012) Species traits, status and cultivar diversity per species were mapped onto the phylogeny to visualise patterns using the R package ‘adephylo’ (Jombart *et al*. 2010) **[see Supporting Information—Fig. S2]**. We used the *D* statistic (Fritz and Purvis 2010) to test for phylogenetic signal and strength of binary traits. This method tests whether traits are randomly assigned across the phylogeny tips (when *D* equals 0), and whether they are clustered (*D* equals 1) under a Brownian threshold model. We carried out two tests: one for introduction status (introduced/not introduced) across the whole phylogeny; in the second, we used a tree trimmed to include only introduced bamboos and tested invasion status (invasive/not invasive). This was done using the R package Caper with function phylo.d (Orme *et al.* 2012).

*Introduction effort and utility* **:** Many species of bamboo have had cultivars developed for improving their utility and value. We suggest that cultivar diversity associated with species could provide a proxy and quantitative means to measure their popularity and utility. Cultivars are cultivated plant varieties that are developed through selective breeding, genetic manipulations such as polyploidization and hybridization. They are often distinctive, uniform and stable and retain key characteristics when propagated (Brickell *et al.*, 2009). Cultivar diversity likely corresponds with propagation frequency and will, therefore, be an important determinant of the probability of introduction, as well as invasion success.

As there is no officially accredited list of bamboo cultivars, we used the list compiled by Ohrnberger (1999) based on the 1995 International Code of Nomenclature for Cultivated Plants (ICNCP). To assess the relationship between introduction status and the number of cultivars developed we used a generalized linear model with a Poisson error structure with number of cultivars as the response variable and status as a predictor variable. As a proxy of introduction effort, we used the number of regions into which a species has been introduced. We tested for this using a generalized linear model with a Poisson error structure with the number of regions a species has been introduced to as a predictor variable and the number of regions a species is invasive in as a response variable.

## Results

### Inventory of species and distribution

Our final list of bamboo species contained 1662 species representing 121 genera, with native species distributed across 122 countries and distinct islands/regions.

### Dissemination and status

**Figure 1:**
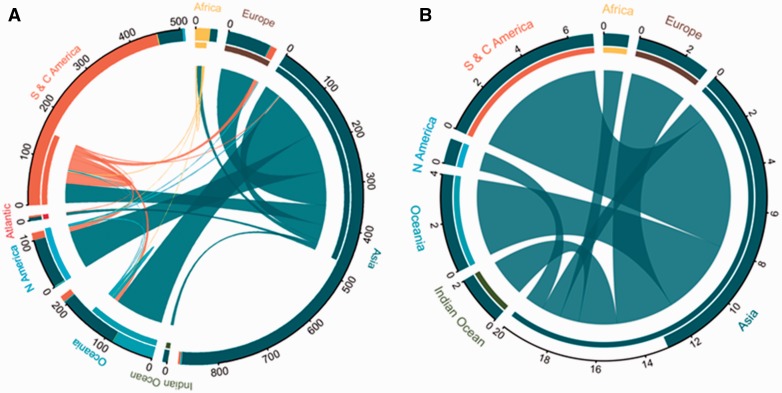
Connectivity plots indicating the transfer of (A) introduced species and (B) invasive species of bamboos around the world relative to their native region. The thickness of internal lines connecting regions correspond to the diversity (number) of species moved. The outer inset bar graph shows the total count of species in that region (by status), and the inner bar graph represents the flow to and from that region. Regions are colour coded by label names.

**Figure 2:**
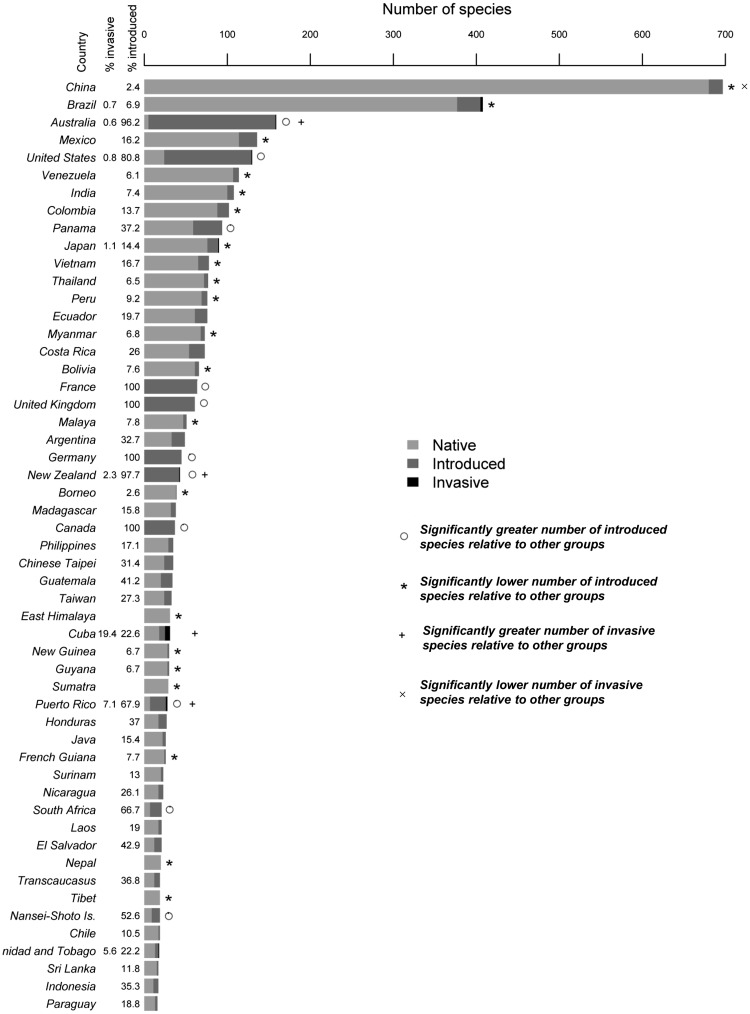
Number of bamboo species found in 52 countries and islands with the highest bamboo richness. Regions with less than 15 species were excluded (135 regions) from the figure. Shading indicates the status of bamboo species in that region (native/introduced/invasive). Significance was calculated using Fisher’s exact tests between numbers of introduced compared with non-introduced species and numbers of invasive compared with non-introduced species across countries.

**Figure 3:**
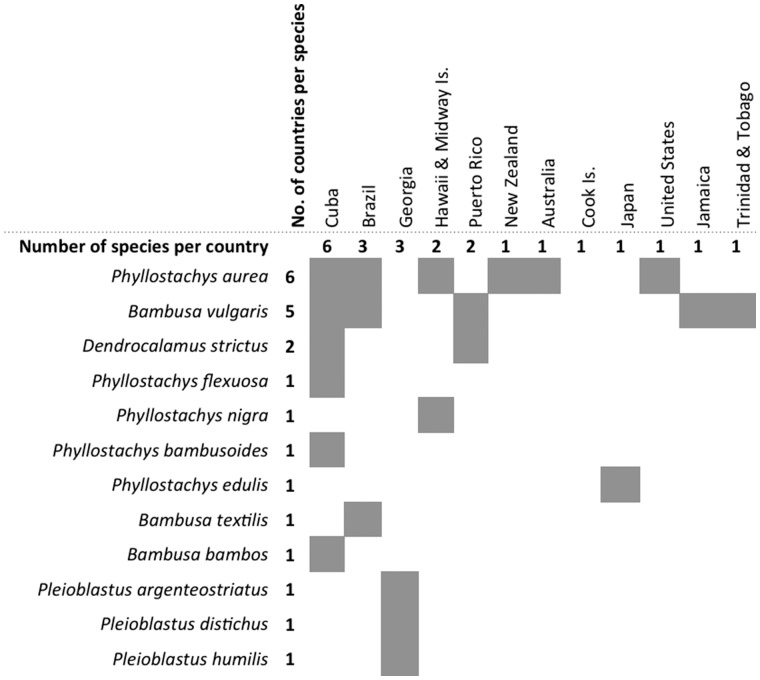
Summary of invasive bamboo species and associated region of invasion.

Two hundred and thirty-two species (14 % of the species in the subfamily) are known to have been introduced outside of their native ranges, with about 5.2 % (12 species) of these introduced species becoming invasive (Fig. 1). However, some regions of the world were markedly over- or under-represented in terms of the number of introduced species (Fig. 2). There were also cases of unknown or disputed native ranges possibly due to a combination of a high degree of introductions and/or lack of reliable records (11 species across 60 countries and regions)**.** Asiatic species have been most widely exported, with Oceania, North America and Europe being the predominant recipients (Fig. 1). All the species reported as invasive are Asiatic. Although South America has a rich native bamboo flora, most movements of these species have been within the continent. We found no evidence of invasive alien bamboos originating from this region. The range of invasive species is shown in Fig. 3.

### Correlates of introduction and invasion

*Morphological traits*: We found all three trait characteristics tested (rhizome form, culm height and culm diameter) to be significantly associated with different stages along the introduction-naturalization-invasion continuum.

For rhizome forms, a significantly higher proportion of introduced species had runner rhizomes (leptomorphs) than clumping rhizomes (pachymorphs), but there was no significant difference in rhizome form for invasive species (Table 2).

**Table 2:** The effect of biogeographic lineage, culm form and underground rhizome form on whether taxa tended to be introduced or become invasive. Each group was tested independently to determine whether species in a particular group or with particular features have been introduced and become invasive significantly more often than other bamboo species. This was done using a Fisher’s exact test comparing the number of introduced versus non-introduced species, and invasive versus non-invasive.

	All	Status					
		Introduced			Invasive		
	*N*	*N*	%	*P*	*N*	%	*P*
Biogeographic lineage							
Temperate woody	500	101	20.2 (16.8–24.0)	0.0067	8	2 (0.9–3.8)	0.022
Paleotropical woody	450	72	16.0 (12.7–19.7)	0.0088	4	1 (0.3–2.7)	1.00
Neotropical woody	300	32	11.0 (7.9–15.0)	0.813	0	–	0.0460
Herbaceous	114	8	7.0 (3.1–13.4)	0.0005	0	–	0.615
Culm form							
Woody	1293	202	16.4 (14.4–18.5)	0.0067	12	1.1 (0.6–1.9)	0.615
Herbaceous	114	7	7.0 (3.1–13.4)	0.0067	0	–	0.615
Underground rhizome form							
Running	331	71	21.4 (16.9–26.4)	0.0018	8	1.6 (0.4–4.1)	0.24
Clumping	860	116	13.5 (11.2–16.0)	0.0018	4	0.7 (0.2–1.6)	0.24

For culm dimensions, there were significant differences between lineages (*F*(3,791)  = 89.65; *P*< 0.001); we, therefore, included lineage affiliation in the analyses below. We found that the average culm diameter for introduced bamboos was significantly greater than for non-introduced bamboos (*R*^2 ^=^ ^0.2687, *F*(5,786)  = 57.75, *P* < 0.001). There was no significant difference in diameter between introduced and invasive species of bamboos in general. Within the paleotropical woody group, species were found to have wider culms relative to other groups. Culm height was greater in the group of introduced species (*P* < 0.001) and for the invasive group (*P* = 0.015), compared with the non-introduced group of species. All woody groups were found to be significantly taller than the herbaceous group (*R*^2 ^=^ ^0.5039, *F*(5, 937) = 190.4, *P* < 0.001).

**Figure 4:**
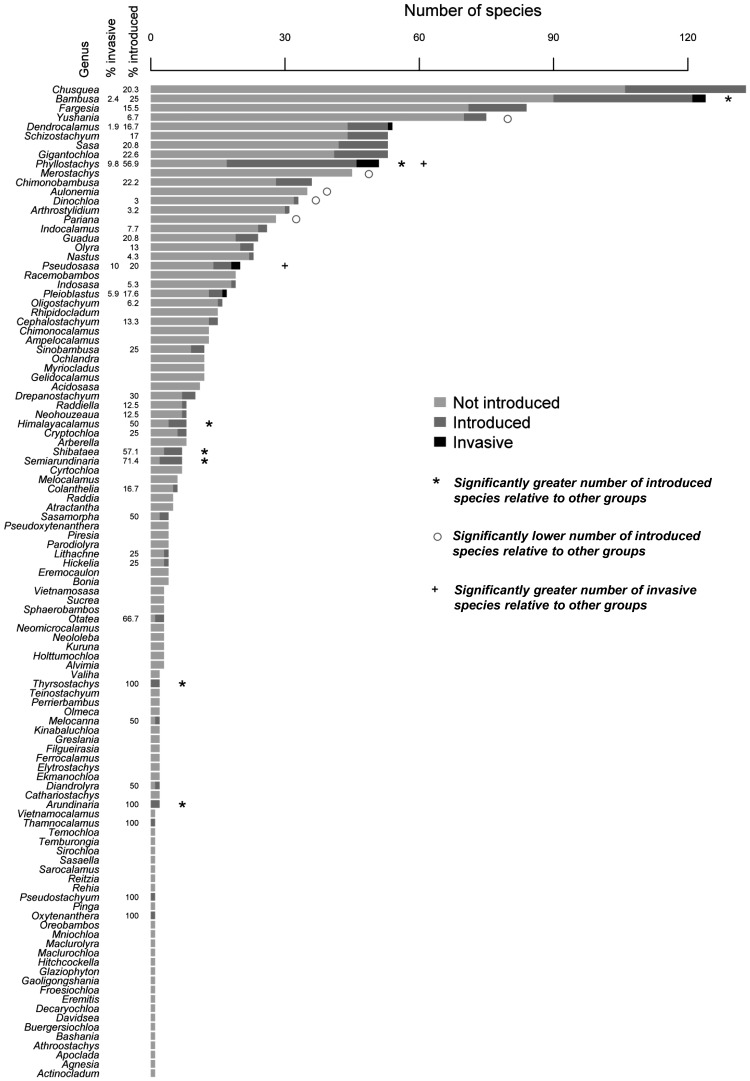
Number of bamboo species found within each genera. Shading indicates the status of the species (not introduced/introduced/invasive). Significance was calculated using Fisher’s exact tests between numbers of introduced compared with non-introduced species and numbers of invasive compared with non-introduced species across genera.

*Taxonomic, geographic and phylogenetic patterns*: At the lineage level, temperate and paleotropical woody bamboo species have been introduced to significantly more countries/regions compared with other groups (Table 2). Herbaceous species had a low proportion of introduced species. Both temperate and paleotropical woody bamboos contained invasive species, yet only temperate woody taxa had a significant proportion of introduced species that have become invasive. At the genus level, there was a significantly (Fisher’s exact test; *P* < 0.05) high proportion of introduced species that belonged to the genera *Arundinaria* (100 %), *Thyrostachys* (100 %), *Semiarundinaria* (71.4 %), *Phyllostachys* (63 %), *Shibateae* (57.1 %), *Himalayacalamus* (50 %) and *Bambusa* (25.6 %) (Fig. 4). *Phyllostachys* (*n* = 5) and *Pseudosasa* (*n* = 2*)* were significant in the number of invasive species, with the remaining invasive species belonging to *Bambusa* (*n*  = 3), *Dendrocalamus* (*n*  = 1) and *Pleioblastus* (*n*  = 1).

With respect to phylogenetic signal, our retrieved phylogeny showed low resolution due to the conservative nature of the *matK* gene. Nevertheless, major and well-supported clades corresponded well with higher-level bamboo taxonomy (e.g. subtribe) and known biogeography. Of the continuous traits tested, culm height (*K* = 0.097, *P*  = 0.014) had a significant phylogenetic signal using Blomberg’s K statistic; but using Pagel’s *λ* both culm height (*λ* = 0.251, *P* < 0.001) and culm diameter (*λ* = 0.418, *P* < 0.001) were significant. For our binary status traits, we found a random pattern for introduction status (*D* = 0.96, *p_rand _ =_ _*0.273*, P_BM _ =_ _*0.00) and for invasion status (D = 1.24, *p_rand _ =_ _*0.77*, P_BM _ =_ _*0.00).

*Introduction effort and utility* **:** We found strong evidence that cultivar diversity was associated with introduction status. Species with more cultivars were significantly more likely to have been introduced (*b= *3.56 ± 0.277, *P*< 0.001) and have become invasive (*b*= 5.89 ± 0.313, *P*< 0.001). Compared with introduced species, invasive species had a greater number of cultivars (*b* = 2.32 ± 0.181, *P* < 0.001), and non-introduced species had significantly fewer cultivars (*b*=−3.56 ± 0.298, *P* < 0.001). Further, we found that the number of regions a species was invasive to be positively and significantly correlated with the number of regions to which a species has been introduced (Poisson GLM: *b*  = 1.02 ± 0.090, *P* < 0.001).

## Discussion

Bamboo species have had a long history of introductions and are now commonly found around the world (Figs 1A and 2) but only a few (12) species are invasive (Fig. 3). As predicted, the movement of bamboos is, however, far from complete and the selection and distribution of species has not been random. We identified three main factors that appear to have influenced patterns of introduction and invasion: introduction effort, propagation of species and selection of traits. Each of these is discussed below and we conclude with an assessment of the current extent of bamboo invasion and expansion of some taxa in their native ranges.

### Introduction effort

Introduction effort, or propagule pressure, has consistently been linked with successful invasions as greater numbers of propagules and more frequent introductions mean higher probabilities of invasion (Colautti *et al.* 2006; Lockwood *et al.* 2005; Von Holle and Simberloff 2005). The positive correlation of propagule pressure and invasion success has been observed in many taxa including birds (Duncan 1997; Veltman *et al.* 1996), mammals (Crowell 1973; Forsyth *et al.* 2004) and aquatic species (Colautti 2005; Duggan *et al.* 2006). This is notable with intentional introductions, such as the case with many ornamental (Dehnen-Schmutz and Touza 2008) and cultivated agricultural (Pyšek *et al.* 2006) plants. We found a clear link between introduction effort and invasiveness in bamboos. Although it was not possible to measure propagule pressure directly, species that had been more widely disseminated were much more likely to have become invasive.

Historical activities in the native range have also played an important role in influencing introduction effort. For example, the local propagation and use of native species may increase the chance of a species becoming established after introductions (Forcella and Wood 1984, Lockwood *et al.* 2005, Pyšek *et al.*2009*a*, *b*). Woody bamboos, in particular, have long been used as a harvested forest resource in regions where they are native (Lobovikov *et al.* 2007). We found that woody bamboos from Asia have been introduced much more often than species from other regions, and all invasive bamboos are native to Asia. This may be explained by an extensive history of active cultivation of woody bamboos around the continent which has promoted the movement of a subset of species (Scurlock *et al.* 2000; Yuming *et al.* 2004; Yuming and Chaomao 2010). Notably in China, bamboo has been widely used for millennia (Li and Kobayashi 2004). Bamboos have shaped the history of this region and they are now an ingrained cultural and economic aspect of many Asian societies. This would have profoundly influenced the way bamboos from this region have been distributed to other parts of the world.

By comparison, the exploitation of bamboo resources in South and Central America, regions also rich in native bamboo species (roughly 32 % species; 530 species), has been historically limited to local and small-scale usage as a forest resource, and, to a lesser extent, as a cultivated crop (Londoño 1998). The number of exported species (or propagation with regards to cultivars) has been low compared with Asiatic species, with the movements being mostly within the continent (Fig. 1A). If these patterns continue, it is likely that future introductions will continue to come from Asia, although there might be significant untapped potential in bamboos from the Americas (Li and Kobayashi 2004).

We found strong selection bias, and, therefore, taxonomic selectivity, for the mostly Asian genera *Bambusa* and *Phyllostachys*. Both genera harbour a high number of invasive species (relative to other bamboo genera) and have been extensively introduced around the world (Fig. 4). *Phyllostachys* is a highly utilized temperate woody genus (59 species) from Asia, mostly central China. More than 50 % of species in this genus have been moved outside of their native ranges (the highest proportion of any bamboo genus), and six species are listed as invasive. *Bambusa*, a paleotropical woody genus, is also highly utilized and is the second largest bamboo genus (149 species). At least 25 % of species in the genus have been introduced to areas outside their natural ranges, and three species have become invasive. Of these, *B. vulgaris* is the most widely distributed species (123 countries); indeed it deserves the title of ‘the most common bamboo in the world’ (Ferrelly 1984). The introduction of *B. vulgaris* to many tropical islands in the Pacific and the Caribbean by early shipping trade routes has left a legacy of naturalized populations (Pacific Island Ecosystems at Risk 2011; O’Connor *et al.* 2000; Rashford 1995).

**Figure 5:**
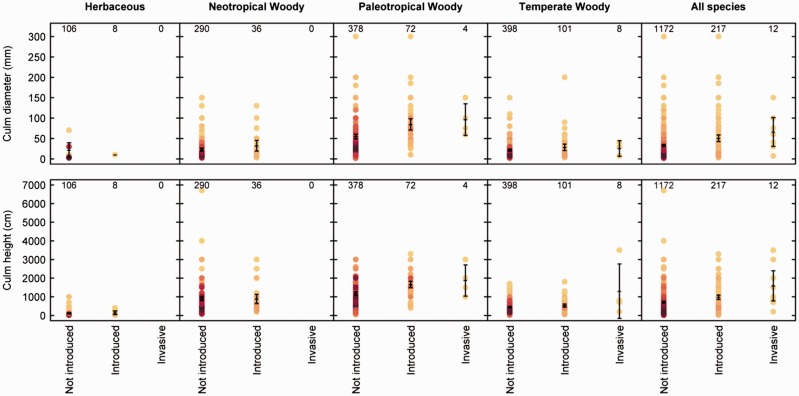
Culm diameter (mm) and culm height (cm) of bamboo species (error bars indicate 95 % confidence intervals) across lineages, and grouped by status. Shading indicates the number of species at each point, with lighter yellow representing less species and darker red shades representing many species. Numbers at the top of each plot indicate the number of species (in which data were available) for the corresponding status group.

### Propagation of species

The fact that some bamboo taxa have been introduced much more widely than others is similar to the patterns observed in other plant groups where there has been a clear bias for species with traits associated with human-usage (Moodley *et al.* 2013; Novoa *et al.* 2015). Species suited for ornamental and agricultural purposes have a higher degree of introduction effort. The horticulture trade in particular has been consistently identified as a major introduction pathway for invasive plants (Dehnen-Schmutz and Touza 2008). Aspects of the industry have been found to be good indicators of risk. For example, increased market availability of species and lower prices of seeds were found to increase the invasion success of species traded in the British horticultural market (Dehnen-Schmutz and Touza 2008).

Drew *et al.* (2010) argued that the horticultural industry is driven by a demand for novel and exotic species, but that there is also a demand for more robust (i.e. with higher stress tolerance) plants for easy maintenance. As the development of cultivars has helped the industry meet some of these demands, cultivar diversity likely reflects the utility (and market demand) of species for horticulture or cultivation. In the case of bamboos, where there has been a consistent and long history of propagation and distribution of plants for horticulture (ornamental plants, landscape improvement, erosion control, etc.) and agroforestry (construction material, crafts, paper pulp, fuel), we expected that the movement of bamboos would be partially influenced by popularity of certain species (Lobovikov *et al.* 2007; Rashford 1995). We found that greater cultivar diversity of species was strongly correlated with the frequency of introductions, and even more so with invasions. We also noted that our list of cultivars were all species of Asian origin, providing further support for the view that historical cultivation of species in this region has been a key determinant for their global export.

Although we did not measure the market preferences directly, cultivar diversity also likely reflects aspects of demand and can help reveal insights into the market preference for certain species. Species that are more widely traded and utilised will have had more efforts made to develop cultivars and vice versa, supporting the notion that market preferences are a key driver of introduction effort with bamboos, as is the case with other economically valuable plant taxa. As far as we know, the link between cultivar development and utility of a species with respect to increasing the probability of introduction and invasions has not been explored for other plant groups.

### Selection of traits

Horticulture directly facilitates the movement of species, but it also provokes the selection of certain traits that can increase establishment and the invasion potential of propagules once introduced (Anderson *et al.* 2006; Dehnen-Schmutz and Touza 2008; Kowarik 2003; Mack 2000; Martínez-Ghersa and Ghersa 2006). Linking traits to the success of invasive species has been a strong focus of invasion science and many studies have revealed generalities across many taxonomic groups. Production of large numbers of seeds, fast growth rates and large plant size are some examples of traits positively associated with invasiveness (Cadotte and Lovett-Doust 2001; Pyšek and Richardson 2007; Van Kleunen *et al.* 2010).

We found that traits likely related to economic benefits are important in bamboos. Culm attributes were associated with the status of species—whether they had been introduced and were invasive; in particular there was an over-representation of introduced and invasive species with greater dimensions. This may be because the culm is a valuable aspect of the plant, and there has been an incentive to select for bigger bamboos to increase production of woody biomass and in general produce larger poles (Kleinhenz and Midmore 2001). However, culm traits did not explain why Asiatic species have been more introduced (and become invasive) than bamboos from other parts of the world. We found that neotropical woody bamboos (of South and Central American origin) were similar to woody bamboo groups in terms of size. Other traits that are important for bamboo as a construction material, which we were unable to test, include culm wall thickness, culm flexibility and internode length.

We expected that the type of clonal growth in bamboos would be an important determinant of invasiveness because bamboos rarely proliferate sexually. It is often suggested in the literature that species that produce long rhizomes (i.e. runner species) are more aggressive than species that produce short rhizomes (RHS 2015). However, we found that both running and clumping species have become invasive. Therefore, the pattern of clonal growth did not clearly separate invasive from non-invasive species and other factors such as human usage, propagule pressure and residency time, need to be considered in any discussion of invasiveness in bamboos.

Species belonging to the genus *Phyllostachys* are most often referenced regarding their ability to spread widely due to fast growth rates and extensive sympodial systems of rhizomes, features which can lead to the formation of monocultures (Isagi and Torii 1997; Suzaki and Nakatsubo 2001). The formation of dense stands can result in a decline in biodiversity through the exclusion of native species (Huai *et al.* 2010; Okutomi *et al.* 1996; ShangBin *et al.* 2013; Yang *et al.* 2008). *Phyllostachys* species have also been shown to invade on a more localised scale, such as in horticultural garden settings (Royal Horticultural Society 2015). In the United States, *Phyllostachys* species (typical examples being *P. aurea, P. aureosulcata*, and *P. edulis*) are distributed and planted as popular ornamental and garden screening plants. However, perhaps due to lack of management and knowledge in maintaining the underground rhizome system, there are reports of populations that have escaped and become naturalized to the extent that they have been shown to occupy 71 588 acres of forests in the US (Miller *et al.* 2008). *Phyllostachys* can also cause a nuisance in urban areas (Connecticut Invasive Plants Council 2011; Joint Standing Committee Hearings 2013). Reported issues in urban areas include structural damage to property from emerging shoots, colonization of gardens and neighbouring land, difficulty and high costs of removing populations due to robust root systems (Joint Standing Committee Hearings 2013). There have been moves to regulate, at the county and state level, the planting and sale of running species (Joint Standing Committee Hearings 2013). With increasing examples of issues surrounding the planting of *Phyllostachys* species, it is likely that other temperate bamboos with similar growth habits and uses will cause similar problems.

### Expansion in the native range

Aspects of the native range have been found to influence the invasiveness of species. For example, species originating from regions with high phylogenetic diversity are more likely to be successful invaders, perhaps because they have more competitive traits (Fridley and Sax 2014). All invasive bamboos originated from Asia, but there was no evidence of a significant phylogenetic signal indicating a particular lineage or clade of bamboo that may be a source for invasive species. This suggests that other factors such as human-mediated usage are more important in explaining invasiveness. However, the corollary of the above observation is that areas with low species richness are likely to be highly invasible (Fridley and Sax 2014). In terms of recipient regions, we did find that the majority (8 out of 12) of the areas where bamboo invasions were recorded were islands (areas of low general native plant diversity and specifically low native bamboo diversity).

Another important factor associated with phylogenetic diversity and invasiveness was the size of the range of species. Species with larger native ranges tend to have greater invasion success, because they possess traits that have facilitated establishment over a wide range of environmental conditions (e.g. Moodley *et al.* 2013; Novoa *et al.* 2014; Pyšek *et al.*2009*a*, *b*). Range size has also been manipulated by human-usage, as many species have been moved and cultivated beyond the extent of their native provenance. We were unable to account for native range size as delimiting ranges for bamboos was difficult, especially in Asia where there has been extensive exchange and cultivation of species over millennia (Lobovikov 2005; Yuming *et al.* 2004). We found many records for the movement of Asiatic species to other continents, but much less information on within-continent movements. For example, Moso bamboo (*Phyllostachys edulis* syn. *P. pubescens*), one species of about 583 native to China, has become widespread (both through natural spread and cultivation) and is estimated to make up 80 % of bamboo cover (5 million ha) across the country (Bowyer *et al.* 2014). Its distribution is still increasing, in part due to extensive plantings but also due to disturbances in mixed forests (Gagnon and Platt 2008) that have facilitated its increased abundance and dominance in some vegetation types (Huai *et al.* 2010; Rother *et al.* 2016; Song *et al.* 2015; ShangBin *et al*; 2013; Tokuoka *et al.* 2015, Yang *et al.* 2008; Xu *et al* 2015).

In general, expansion and weedy behaviour of plants in their native range has been shown to be a good indicator of invasive potential (e.g. Richardson and Bond 1991). As past introductions of bamboos have favoured a certain set of species from particular regions, there is significant potential for bamboos in other parts of the world such as South America to be utilised in the future. Such species that have been identified as being highly competitive and weedy in native regions have the potential to become invasive in new areas given the opportunity, and should be carefully evaluated for future introductions. Some examples of bamboos that are found to be weedy and have had impacts in their native ranges are *Pleioblastus arenteostriatus* (syn. *P. chino*; Kobayashi *et al.* 1999; Tokuoka *et al.* 2015), *Fargesia nitida* (Wang *et al.* 2012) and *Sasa chartacea* (Tomimatsu *et al.* 2011) in East Asia*. Ochlandra travancorica* (Dutta and Reddy 2016) and *Melocanna baccifera* (Majumdar *et al.* 2015) from India, and *Guadua tagoara* (Rother *et al.* 2016) and *Guadua paraguayana* (Galvão *et al.* 2012) from South America have not been widely moved outside of their native ranges but, given the observed weedy tendencies of these species in their native ranges, they could pose risks if future introductions were to occur.

Without accurate records on the original ranges of many taxa, it is difficult to comment on the rate of spread and the extent of invasions. We suspect that invasions of some species may have gone unnoticed. This is due to scant information on the native provenance in some regions, and problems with identifying some bamboo species. This is the case where some species are widely dispersed at the continental level and are assumed to be native while they may well be introduced in parts of their current range.

### Extent of invasions

Overall, we found few invasive species of bamboos (0.7 % of taxa) despite the diversity, high rate of dissemination and utilization of various species globally; we had expected this number to be higher. The low number of invasive bamboos is in marked contrast with other taxa within the grass family, which have been noted for containing a high concentration of invasive species (studies estimate between 6 and 10 %; Pyšek 1998; Visser *et al.* 2016). Bamboos seem to be an exception in the group. Some of the most extensive invaders in the grass family are large-statured woody grasses, notably *Arundo donax* and *Phragmites australis* (D'Antonio *et al.* 2010; Lambert *et al.* 2010). These invasive woody grasses mostly rely on asexual means for spreading via the rhizome systems like many bamboos (Nadgauda *et al.* 1990). There is scope to investigate such mechanisms in explaining the ability of some large-statured woody grass species to be widespread invaders and why this appears not to be the general case with bamboos.

When compared with other plant taxa outside of the grass family, bamboos have a similarly low occurrence of invasive species; in the group of trees and shrubs it was found that between 0.5 % and 0.7 % of the global pool of species had become invasive (Richardson and Rejmánek 2011), and for the families of Proteaceae (Moodley *et al.* 2013), Araceae (Moodley *et al.* 2016) and Cactaceae (Novoa *et al.* 2015), 2 %, 0.5 % and 3 % are invasive, respectively.

We discounted invasions in 26 regions (including those involving three additional species) as references could not be verified or were inaccessible. We suspect that the listing of some bamboos as invasive may be unwarranted (or inflated). This is the case with *Dendrocalamus strictus*, for which it was difficult to disentangle the rate of spread versus impacts, as there was not an explicit distinction in many references **[see Supporting Information—Table S3].** In many cases, a long history of planting of bamboos gave the appearance of a prolific, spreading population, whereas the expansion of the population has in fact been minimal or non-existent (O’Connor *et al.* 2000). For this reason, it is important that standardized and measurable criteria be adopted for defining what ‘invasive’ means for bamboos.

## Conclusions

Our results suggest that invasiveness in bamboo species is currently more a function of which species have been moved by humans and for what purposes than of inherent differences between species. Certain taxa, for historical and geographical reasons, have rarely been introduced. In particular, native South American bamboos have not yet been widely disseminated. Such taxa might hold promise for future utilisation, and could become invasive. By contrast, past introductions (especially from Asia) have radically rearranged the global distribution of some bamboo species, and new trends in the drivers of introductions are rapidly changing the dimensions in this natural experiment in biogeography. The emergence of large-scale bamboo plantations in new regions of the world represents a fascinating new stage in the bamboo story. There is an urgent need for science-based guidelines to minimize invasion risks.

## Sources of Funding

This work was supported by the South African National Department of Environment Affairs through its funding of the South African National Biodiversity Institute Invasive Species Programme, the DST-NRF Centre of Excellence for Invasion Biology, and the National Research Foundation of South Africa (Grant 85417 to D.M.R.).

## Contributions by the Authors

S.C, J.R.U.W and D.M.R conceived the idea. S.C compiled the data. V.V. contributed to analysing and visualizing data for final publication. J.J.L.R. assembled the phylogeny. M.V. provided the GrassBase database. S.C. led the writing of the manuscript with inputs from all co-authors.

## Conflicts of Interest

None declared.

## Supplementary Material

Supplementary Data
