# Supplementary material for: The global distribution of bamboos: assessing correlates of introduction and invasion
Source: AoB Plants. 2016 Dec 23;9(1):plw078. doi: 10.1093/aobpla/plw078 (PMC5499700; doi:10.1093/aobpla/plw078)
Supplement: Supplementary Data [file plw078_Supp.docx]

**Table S1.** List of Bambusoideae species (1662 species) based on accepted taxa from Kew’s GrassBase (http://www.kew.org/data/grasses-syn.html) updated to include recent literature on new species and other changes in classification that have been published up until September 2015, described at the generic level in Kellogg (2015). Updates were contributed by bamboo taxanomy specialist Lynn G. Clark (Iowa State Univeristy) and grass taxanomy specialist, Maria S. Vorontsova. *232 species have been Introduced outside of their native range (numbers following species indicate the number of introduced regions), **†**12 species are referenced as being invasive,^?^11 species have unknown or disputed native ranges. Note that the list does not include contemporary hybrids.

*Acidosasa breviclavata*

*Acidosasa brilletii*

*Acidosasa carinata*

*Acidosasa chienouensis*

*Acidosasa chinensis*

*Acidosasa edulis*

*Acidosasa guangxiensis*

*Acidosasa lingchuanensis*

*Acidosasa nanunica*

*Acidosasa notata*

*Acidosasa purpurea*

*Acidosasa venusta*

*Actinocladum verticillatum*

*Agnesia lancifolia*

*Alvimia auriculata*

*Alvimia gracilis*

*Alvimia lancifolia*

*Ampelocalamus actinotrichus*

*Ampelocalamus breviligulatus*

*Ampelocalamus calcareus*

*Ampelocalamus hirsutissimus*

*Ampelocalamus luodianensis*

*Ampelocalamus melicoideus*

*Ampelocalamus mianningensis*

*Ampelocalamus microphyllus*

*Ampelocalamus naibunensis*

*Ampelocalamus patellaris*

*Ampelocalamus saxatilis*

*Ampelocalamus scandens*

*Ampelocalamus yongshanensis*

*Annamocalamus kontumensis*

*Apoclada simplex*

*Arberella bahiensis*

*Arberella costaricensis*

*Arberella dressleri*

*Arberella flaccida*

*Arberella grayumii*

*Arberella lancifolia*

*Arberella venezuelae*

*Arthrostylidium angustifolium*

*Arthrostylidium auriculatum*

*Arthrostylidium banaoense*

*Arthrostylidium berryi*

*Arthrostylidium canaliculatum*

*Arthrostylidium chiribiquetense*

*Arthrostylidium cubense*

*Arthrostylidium distichum*

*Arthrostylidium ecuadorense*

*Arthrostylidium ekmanii*

*Arthrostylidium excelsum*

*Arthrostylidium farctum*

*Arthrostylidium fimbriatum*

*Arthrostylidium fimbrinodum*

*Arthrostylidium grandifolium*

*Arthrostylidium haitiense*

*Arthrostylidium judziewiczii*

*Arthrostylidium longiflorum*

*Arthrostylidium merostachyoides*

*Arthrostylidium multispicatum*

*Arthrostylidium obtusatum*

*Arthrostylidium pubescens*

*Arthrostylidium punctulatum*

*Arthrostylidium reflexum*

*Arthrostylidium sarmentosum*(1)*

*Arthrostylidium scandens*

*Arthrostylidium schomburgkii*

*Arthrostylidium simpliciusculum*

*Arthrostylidium urbanii*

*Arthrostylidium venezuelae*

*Arthrostylidium virolinense*

*Arthrostylidium youngianum*

*Arundinaria appalachiana*(1)*

*Arundinaria gigantea*(6)*

*Arundinaria tecta*

*Athroostachys capitata*

*Atractantha amazonica*

*Atractantha aureolanata*

*Atractantha cardinalis*

*Atractantha falcata*

*Atractantha radiata*

*Atractantha shepherdiana*

*Aulonemia amplissima*

*Aulonemia aristulata*

*Aulonemia bogotensis*

*Aulonemia boliviana*

*Aulonemia bromoides*

*Aulonemia chimantaensis*

*Aulonemia cincta*

*Aulonemia cochabambensis*

*Aulonemia david-smithii*

*Aulonemia deflexa*

*Aulonemia dinirensis*

*Aulonemia effusa*

*Aulonemia fuentesii*

*Aulonemia glaziovii*

*Aulonemia goyazensis*

*Aulonemia haenkei*

*Aulonemia herzogiana*

*Aulonemia hirtula*

*Aulonemia humillima*

*Aulonemia insignis*

*Aulonemia jauaensis*

*Aulonemia laxa*

*Aulonemia longiaristata*

*Aulonemia longipedicellata*

*Aulonemia madidiensis*

*Aulonemia nitida*

*Aulonemia notata*

*Aulonemia parviflora*

*Aulonemia patriae*

*Aulonemia patula*

*Aulonemia prolifera*

*Aulonemia pumila*

*Aulonemia purpurata*

*Aulonemia queko*

*Aulonemia radiata*

*Aulonemia ramosissima*

*Aulonemia robusta*

*Aulonemia rubraligulata*

*Aulonemia scripta*

*Aulonemia setigera*

*Aulonemia setosa*

*Aulonemia soderstromii*

*Aulonemia subpectinata*

*Aulonemia tremula*

*Aulonemia trianae*

*Aulonemia verrucosa*

*Aulonemia viscosa*

*Aulonemia xerophylla*

*Aulonemia ximenae*

*Aulonemia yanachagensis*

*Bambusa affinis*

*Bambusa alamii*

*Bambusa albolineata*

*Bambusa alemtemshii*

*Bambusa amahussana*

*Bambusa amplexicaulis*(1)*

*Bambusa angustiaurita*

*Bambusa angustissima*

*Bambusa aristata*

*Bambusa arnhemica*

*Bambusa assamica*

*Bambusa aurinuda*

*Bambusa australis*

*Bambusa balcooa*?(16)*

*Bambusa bambos*†(40)*

*Bambusa barpatharica*

*Bambusa basihirsuta*

*Bambusa basihirsutoides*

*Bambusa beecheyana*(6)*

*Bambusa bicicatricata*

*Bambusa binghamii*

*Bambusa blumeana*?(15)*

*Bambusa boniopsis*

*Bambusa brevispicula*

*Bambusa brunneoaciculia*

*Bambusa burmanica*

*Bambusa cacharensis*

*Bambusa cerosissima*

*Bambusa chungii*(1)*

*Bambusa chunii*

*Bambusa clavata*

*Bambusa comillensis*

*Bambusa contracta*

*Bambusa copelandii*

*Bambusa corniculata*

*Bambusa cornigera*

*Bambusa crispiaurita*

*Bambusa dampaeana*

*Bambusa diaoluoshanensis*

*Bambusa dissimulator*(4)*

*Bambusa distegia*

*Bambusa dolichoclada*(3)*

*Bambusa duriuscula*

*Bambusa emeiensis*(1)*

*Bambusa eutuldoides*(2)*

*Bambusa farinacea*

*Bambusa fimbriligulata*

*Bambusa flexuosa*(1)*

*Bambusa fruticosa*

*Bambusa funghomii*

*Bambusa garuchokua*

*Bambusa gibba*

*Bambusa gibboides*

*Bambusa glabrovagina*

*Bambusa glaucophylla*

*Bambusa grandis*

*Bambusa griffithiana*

*Bambusa guangxiensis*

*Bambusa hainanensis*

*Bambusa heterostachya*(1)*

*Bambusa inaurita*

*Bambusa indigena*

*Bambusa insularis*

*Bambusa intermedia*(1)*

*Bambusa jacobsii*

*Bambusa khasiana*

*Bambusa kingiana*(1)*

*Bambusa kyathaungtu*

*Bambusa lako*(2)*

*Bambusa lapidea*

*Bambusa latideltata*

*Bambusa laxa*

*Bambusa lenta*

*Bambusa lineata*

*Bambusa longipalea*

*Bambusa longispiculata*(13)*

*Bambusa macrolemma*

*Bambusa macrotis*

*Bambusa maculata*

*Bambusa majumdarii*

*Bambusa malingensis*(4)*

*Bambusa manipureana*

*Bambusa marginata*

*Bambusa merrillii*

*Bambusa mitis*

*Bambusa mizorameana*

*Bambusa mohanramii*

*Bambusa mollis*

*Bambusa multiplex*?(58)*

*Bambusa mutabilis*

*Bambusa nagalandiana*

*Bambusa nairiana*

*Bambusa nepalensis*

*Bambusa nutans*(3)*

*Bambusa odashimae*(1)*

*Bambusa oldhamii*(21)*

*Bambusa oliveriana*(2)*

*Bambusa ooh*

*Bambusa pachinensis*

*Bambusa pallida*

*Bambusa papillata*

*Bambusa papillatoides*

*Bambusa pervariabilis*(1)*

*Bambusa pierreana*

*Bambusa piscatorum*

*Bambusa polymorpha*(12)*

*Bambusa procera*

*Bambusa prominens*

*Bambusa ramispinosa*

*Bambusa rangaensis*

*Bambusa rectocuneata*

*Bambusa remotiflora*(1)*

*Bambusa riauensis*

*Bambusa rigida*

*Bambusa riparia*(1)*

*Bambusa rongchengensis*

*Bambusa rugata*

*Bambusa rutila*

*Bambusa salarkhanii*

*Bambusa semitecta*

*Bambusa sesquiflora*

*Bambusa sinospinosa*(1)*

*Bambusa sinthana*

*Bambusa solomonensis*

*Bambusa stenoaurita*

*Bambusa subaequalis*

*Bambusa subtruncata*

*Bambusa surrecta*

*Bambusa teres*

*Bambusa textilis*†(12)*

*Bambusa thalawwa*

*Bambusa thorelii*

*Bambusa transvenula*

*Bambusa truncata*

*Bambusa tsangii*

*Bambusa tulda*(14)*

*Bambusa tuldoides*(27)*

*Bambusa utilis*(1)*

*Bambusa valida*

*Bambusa variostriata*(1)*

*Bambusa villosula*

*Bambusa vinhphuensis*

*Bambusa virginalis*

*Bambusa viridis*(5)*

*Bambusa vulgaris*†?(127)*

*Bambusa wenchouensis*

*Bambusa wiesneri*

*Bambusa xiashanensis*

*Bambusa xueana*

*Bashania fargesii*

*Bashania qingchengshanensis*

*Bergbambos tessellata*(3)*

*Bonia amplexicaulis*

*Bonia levigata*

*Bonia parvifloscula*

*Bonia solida*

*Bonia tonkinensis*

*Buergersiochloa bambusoides*

*Cambajuva ulei*

*Cathariostachys capitata*

*Cathariostachys madagascariensis*

*Cephalostachyum burmanicum*

*Cephalostachyum capitatum*

*Cephalostachyum chapelieri*

*Cephalostachyum chevalieri*

*Cephalostachyum flavescens*

*Cephalostachyum langbianense*

*Cephalostachyum latifolium*

*Cephalostachyum mannii*

*Cephalostachyum mindorense*

*Cephalostachyum pallidum*

*Cephalostachyum pergracile*(6)*

*Cephalostachyum perrieri*

*Cephalostachyum scandens*

*Cephalostachyum viguieri*

*Cephalostachyum virgatum*(2)*

*Chimonobambusa angustifolia*(1)*

*Chimonobambusa armata*

*Chimonobambusa arunachalensis*

*Chimonobambusa brevinoda*

*Chimonobambusa callosa*

*Chimonobambusa communis*

*Chimonobambusa convoluta*

*Chimonobambusa damingshanensis*

*Chimonobambusa fansipanensis*

*Chimonobambusa gracilis*

*Chimonobambusa grandifolia*

*Chimonobambusa hejiangensis*(1)*

*Chimonobambusa hirtinoda*

*Chimonobambusa hsuehiana*

*Chimonobambusa jainii*

*Chimonobambusa lactistriata*

*Chimonobambusa leishanensis*

*Chimonobambusa luzhiensis*

*Chimonobambusa macrophylla*(2)*

*Chimonobambusa marmorea*(11)*

*Chimonobambusa metuoensis*

*Chimonobambusa microfloscula*

*Chimonobambusa montigena*

*Chimonobambusa ningnanica*

*Chimonobambusa opienensis*

*Chimonobambusa pachystachys*(1)*

*Chimonobambusa paucispinosa*

*Chimonobambusa puberula*

*Chimonobambusa pubescens*

*Chimonobambusa purpurea*

*Chimonobambusa quadrangularis*(17)*

*Chimonobambusa rigidula*

*Chimonobambusa sichuanensis*

*Chimonobambusa szechuanensis*(1)*

*Chimonobambusa tuberculata*

*Chimonobambusa tumidissinoda*(5)*

*Chimonobambusa unifolia*

*Chimonobambusa utilis*

*Chimonobambusa verruculosa*

*Chimonocalamus baviensis*

*Chimonocalamus burmaensis*

*Chimonocalamus cibarius*

*Chimonocalamus delicatus*

*Chimonocalamus dumosus*

*Chimonocalamus fimbriatus*

*Chimonocalamus gallatlyi*

*Chimonocalamus griffithianus*

*Chimonocalamus longiligulatus*

*Chimonocalamus longispiculatus*

*Chimonocalamus longiusculus*

*Chimonocalamus lushaiensis*

*Chimonocalamus makuanensis*

*Chimonocalamus montanus*

*Chimonocalamus nagalandianus*

*Chimonocalamus pallens*

*Chimonocalamus peregrinus*

*Chimonocalamus tortuosus*

*Chusquea abietifolia*(1)*

*Chusquea acuminata*

*Chusquea acuminatissima*

*Chusquea albilanata*

*Chusquea amistadensis*

*Chusquea andina*

*Chusquea anelytra*

*Chusquea anelytroides*

*Chusquea angusta*

*Chusquea angustifolia*

*Chusquea annagardneriae*

*Chusquea antioquensis*

*Chusquea aperta*

*Chusquea arachniformis*

*Chusquea argentina*

*Chusquea aristata*

*Chusquea aspera*

*Chusquea asymmetrica*

*Chusquea attenuata*

*Chusquea aurea*

*Chusquea baculifera*

*Chusquea bahiana*

*Chusquea bambusoides*(1)*

*Chusquea barbata*

*Chusquea bilimekii*

*Chusquea bradei*

*Chusquea caamanoi*

*Chusquea caparaoensis*

*Chusquea capitata*

*Chusquea capituliflora*

*Chusquea ciliata*

*Chusquea ciliatifolia*

*Chusquea circinata*(2)*

*Chusquea clarkiae*

*Chusquea clemirae*

*Chusquea coronalis*(3)*

*Chusquea cortesii*

*Chusquea costaricensis*

*Chusquea culeou*(5)*

*Chusquea cumingii*(1)*

*Chusquea cylindrica*

*Chusquea decolorata*

*Chusquea deficiens*(2)*

*Chusquea deflexa*

*Chusquea delicatula*

*Chusquea depauperata*

*Chusquea diversiglumis*

*Chusquea dombeyana*

*Chusquea egluma*

*Chusquea elata*

*Chusquea enigmatica*

*Chusquea erecta*

*Chusquea exasperata*

*Chusquea falcata*

*Chusquea fasciculata*

*Chusquea fendleri*

*Chusquea fernandezia*

*Chusquea fimbriligulata*

*Chusquea floribunda*

*Chusquea foliosa*(2)*

*Chusquea galeottiana*

*Chusquea gigantea*

*Chusquea glauca*

*Chusquea glomerata*

*Chusquea gracilis*

*Chusquea grandiflora*

*Chusquea guirigayensis*

*Chusquea hatschbachii*

*Chusquea heterophylla*(1)*

*Chusquea huantensis*

*Chusquea ibiramae*

*Chusquea inamoena*

*Chusquea juergensii*(1)*

*Chusquea laegaardii*

*Chusquea lanceolata*(1)*

*Chusquea latifolia*

*Chusquea lehmannii*(2)*

*Chusquea leonardiorum*

*Chusquea leptophylla*

*Chusquea liebmannii*

*Chusquea ligulata*

*Chusquea linearis*(3)*

*Chusquea londoniae*

*Chusquea longifolia*

*Chusquea longiligulata*

*Chusquea longipendula*

*Chusquea longiprophylla*

*Chusquea longispiculata*

*Chusquea lorentziana*(1)*

*Chusquea loxensis*

*Chusquea maclurei*

*Chusquea macrostachya*

*Chusquea maculata*

*Chusquea magnifolia*

*Chusquea matlatzinca*

*Chusquea mayrae*

*Chusquea meyeriana*(1)*

*Chusquea microphylla*

*Chusquea mimosa*

*Chusquea mirabilis*

*Chusquea mollis*

*Chusquea montana*(1)*

*Chusquea mulleri*

*Chusquea multiramea*

*Chusquea nana*

*Chusquea nelsonii*

*Chusquea neurophylla*

*Chusquea nobilis*

*Chusquea nudiramea*

*Chusquea nutans*(2)*

*Chusquea oligophylla*

*Chusquea oxylepis*

*Chusquea pallida*

*Chusquea paludicola*

*Chusquea patens*

*Chusquea perligulata*

*Chusquea perotensis*

*Chusquea peruviana*

*Chusquea petiolata*

*Chusquea picta*

*Chusquea pinifolia*(1)*

*Chusquea pittieri*

*Chusquea pohlii*

*Chusquea polyclados*

*Chusquea pubispicula*

*Chusquea pulchella*

*Chusquea purdieana*

*Chusquea quila*

*Chusquea ramosissima*(2)*

*Chusquea renvoizei*

*Chusquea repens*(1)*

*Chusquea rigida*

*Chusquea riosaltensis*

*Chusquea robusta*

*Chusquea scabra*

*Chusquea scandens*(4)*

*Chusquea sclerophylla*

*Chusquea sellowii*

*Chusquea serpens*

*Chusquea serrulata*(2)*

*Chusquea silverstonei*

*Chusquea simpliciflora*(1)*

*Chusquea smithii*

*Chusquea sneidernii*

*Chusquea spadicea*

*Chusquea spathacea*

*Chusquea spectabilis*

*Chusquea spencei*(13)*

*Chusquea spicata*

*Chusquea straminea*

*Chusquea stuebelii*

*Chusquea subtessellata*

*Chusquea subtilis*

*Chusquea subulata*

*Chusquea sulcata*

*Chusquea talamancensis*

*Chusquea tarmensis*

*Chusquea tenella*

*Chusquea tenuiglumis*

*Chusquea tessellata*(1)*

*Chusquea tomentosa*

*Chusquea tonduzii*(1)*

*Chusquea tovari*

*Chusquea tuberculosa*

*Chusquea uliginosa*

*Chusquea uniflora*

*Chusquea urelytra*

*Chusquea valdiviensis*(1)*

*Chusquea villosa*

*Chusquea virgata*

*Chusquea vulcanalis*

*Chusquea wilkesii*

*Chusquea windischii*

*Chusquea yungasensis*

*Colanthelia burchellii*

*Colanthelia cingulata*

*Colanthelia distans*

*Colanthelia intermedia*

*Colanthelia lanciflora*

*Colanthelia macrostachya*

*Colanthelia rhizantha*(1)*

*Cryptochloa capillata*(1)*

*Cryptochloa concinna*

*Cryptochloa decumbens*

*Cryptochloa dressleri*

*Cryptochloa soderstromii*

*Cryptochloa strictiflora*(6)*

*Cryptochloa unispiculata*

*Cryptochloa variana*

*Cyrtochloa fenixii*

*Cyrtochloa hirsuta*

*Cyrtochloa luzonica*

*Cyrtochloa major*

*Cyrtochloa mindoroensis*

*Cyrtochloa puser*

*Cyrtochloa toppingii*

*Davidsea attenuata*

*Decaryochloa diadelpha*

*Dendrocalamus asper*?(11)*

*Dendrocalamus bacthaiensis*

*Dendrocalamus bambusoides*

*Dendrocalamus barbatus*(1)*

*Dendrocalamus bengkalisensis*

*Dendrocalamus birmanicus*

*Dendrocalamus brandisii*(2)*

*Dendrocalamus buar*

*Dendrocalamus calostachyus*(1)*

*Dendrocalamus cauhaiensis*

*Dendrocalamus cinctus*

*Dendrocalamus collettianus*

*Dendrocalamus detinens*

*Dendrocalamus dumosus*

*Dendrocalamus elegans*

*Dendrocalamus exauritus*

*Dendrocalamus farinosus*

*Dendrocalamus fugongensis*

*Dendrocalamus giganteus*?(15)*

*Dendrocalamus hait*

*Dendrocalamus hamiltonii*(1)*

*Dendrocalamus hirtellus*

*Dendrocalamus hookeri*

*Dendrocalamus jianshuiensis*

*Dendrocalamus khoonmengii*

*Dendrocalamus latiflorus*?(11)*

*Dendrocalamus liboensis*

*Dendrocalamus longispathus*(1)*

*Dendrocalamus macroculmis*

*Dendrocalamus maiensis*

*Dendrocalamus manipureanus*

*Dendrocalamus membranaceus*(4)*

*Dendrocalamus menglongensis*

*Dendrocalamus merrillianus*

*Dendrocalamus messeri*

*Dendrocalamus minor*

*Dendrocalamus multiflosculus*

*Dendrocalamus nhatrangensis*

*Dendrocalamus nianhei*

*Dendrocalamus nudus*

*Dendrocalamus pachystachyus*

*Dendrocalamus parishii*

*Dendrocalamus parvigemma*

*Dendrocalamus peculiaris*

*Dendrocalamus pendulus*

*Dendrocalamus poilanei*

*Dendrocalamus pulverulentus*

*Dendrocalamus rugosiglumis*

*Dendrocalamus sahnii*

*Dendrocalamus sang*

*Dendrocalamus semiscandens*

*Dendrocalamus sericeus*

*Dendrocalamus sikkimensis*

*Dendrocalamus sinicus*

*Dendrocalamus sinuatus*

*Dendrocalamus somdevae*

*Dendrocalamus strictus*†(27)*

*Dendrocalamus suberosus*

*Dendrocalamus taybacensis*

*Dendrocalamus tibeticus*

*Dendrocalamus tomentosus*

*Dendrocalamus triramus*

*Dendrocalamus tsiangii*

*Dendrocalamus velutinus*

*Dendrocalamus wabo*

*Dendrocalamus xishuangbannaensis*

*Dendrocalamus yentuensis*

*Dendrocalamus yunnanicus*

*Diandrolyra bicolor*(1)*

*Diandrolyra pygmaea*

*Diandrolyra tatianae*

*Didymogonyx geminatum*(1)*

*Didymogonyx longispiculatum*

*Dinochloa acutiflora*

*Dinochloa alata*

*Dinochloa albociliata*

*Dinochloa andamanica*

*Dinochloa aopaensis*

*Dinochloa barbata*

*Dinochloa cordata*

*Dinochloa darvelana*

*Dinochloa dielsiana*

*Dinochloa diffusa*

*Dinochloa elmeri*

*Dinochloa erecta*

*Dinochloa glabrescens*

*Dinochloa hirsuta*

*Dinochloa kostermansiana*

*Dinochloa luconiae*

*Dinochloa macclellandii*

*Dinochloa malayana*(1)*

*Dinochloa matmat*

*Dinochloa morowaliensis*

*Dinochloa nicobariana*

*Dinochloa obclavata*

*Dinochloa oblonga*

*Dinochloa orenuda*

*Dinochloa palawanensis*

*Dinochloa petasiensis*

*Dinochloa prunifera*

*Dinochloa pubiramea*

*Dinochloa robusta*

*Dinochloa scabrida*

*Dinochloa scandens*

*Dinochloa sepang*

*Dinochloa sipitangensis*

*Dinochloa sublaevigata*

*Dinochloa trichogona*

*Dinochloa truncata*

*Dinochloa utilis*

*Drepanostachyum ampullare*

*Drepanostachyum annulatum*

*Drepanostachyum falcatum*(5)*

*Drepanostachyum fractiflexum*

*Drepanostachyum intermedium*(1)*

*Drepanostachyum khasianum*(1)*

*Drepanostachyum kurzii*

*Drepanostachyum membranaceum*

*Drepanostachyum merretii*

*Drepanostachyum polystachyum*

*Drepanostachyum semiorbiculatum*

*Drepanostachyum stoloniforme*

*Ekmanochloa aristata*

*Ekmanochloa subaphylla*

*Elytrostachys clavigera*

*Elytrostachys typica*

*Eremitis afimbriata*

*Eremitis magnifica*

*Eremitis parviflora*

*Eremocaulon amazonicum*

*Eremocaulon asymmetricum*

*Eremocaulon aureofimbriatum*

*Eremocaulon capitatum*

*Fargesia acuticontracta*

*Fargesia adpressa*

*Fargesia albocerea*

*Fargesia altior*

*Fargesia angustissima*(1)*

*Fargesia apicirubens*

*Fargesia boliana*

*Fargesia brevipes*

*Fargesia brevissima*

*Fargesia caduca*

*Fargesia canaliculata*

*Fargesia circinata*

*Fargesia communis*

*Fargesia concinna*

*Fargesia conferta*

*Fargesia contracta*

*Fargesia cuspidata*

*Fargesia daminiu*

*Fargesia declivis*

*Fargesia decurvata*

*Fargesia denudata*(1)*

*Fargesia dracocephala*(4)*

*Fargesia dulcicula*

*Fargesia dura*

*Fargesia edulis*

*Fargesia elegans*

*Fargesia emaculata*

*Fargesia emeryi*

*Fargesia exposita*

*Fargesia extensa*

*Fargesia fansipanensis*

*Fargesia farcta*

*Fargesia ferax*(1)*

*Fargesia frigida*

*Fargesia fungosa*(3)*

*Fargesia funiushanensis*

*Fargesia glabrifolia*

*Fargesia gongshanensis*

*Fargesia grossa*

*Fargesia hackelii*

*Fargesia hainanensis*

*Fargesia hsuehiana*

*Fargesia huizensis*

*Fargesia hygrophila*

*Fargesia jiulongensis*

*Fargesia lincangensis*

*Fargesia longiuscula*

*Fargesia lushuiensis*

*Fargesia macclureana*(1)*

*Fargesia mairei*

*Fargesia mali*

*Fargesia melanostachys*

*Fargesia murielae*(6)*

*Fargesia nitida*(8)*

*Fargesia nujiangensis*

*Fargesia obliqua*

*Fargesia orbiculata*

*Fargesia papyrifera*

*Fargesia pauciflora*

*Fargesia perlonga*

*Fargesia pleniculmis*

*Fargesia plurisetosa*

*Fargesia porphyrea*

*Fargesia praecipua*

*Fargesia qinlingensis*

*Fargesia robusta*(4)*

*Fargesia rufa*(2)*

*Fargesia sagittatinea*

*Fargesia scabrida*(1)*

*Fargesia schmidiana*

*Fargesia semicoriacea*

*Fargesia similaris*

*Fargesia solida*

*Fargesia spathacea*(5)*

*Fargesia stenoclada*

*Fargesia strigosa*

*Fargesia subflexuosa*

*Fargesia sylvestris*

*Fargesia tenuilignea*

*Fargesia ungulata*

*Fargesia utilis*(3)*

*Fargesia vicina*

*Fargesia weiningensis*

*Fargesia wuliangshanensis*

*Fargesia yajiangensis*

*Fargesia yuanjiangensis*

*Fargesia yulongshanensis*

*Fargesia yunnanensis*

*Fargesia zayuensis*

*Ferrocalamus fibrillosus*

*Ferrocalamus rimosivaginus*

*Ferrocalamus strictus*

*Filgueirasia arenicola*

*Filgueirasia cannavieira*

*Fimbribambusa horsfieldii*

*Fimbribambusa microcephala*

*Froesiochloa boutelouoides*

*Gaoligongshania megalothyrsa*

*Gelidocalamus albopubescens*

*Gelidocalamus annulatus*

*Gelidocalamus dongdingensis*

*Gelidocalamus kunishii*

*Gelidocalamus latifolius*

*Gelidocalamus longiinternodus*

*Gelidocalamus multifolius*

*Gelidocalamus rutilans*

*Gelidocalamus solidus*

*Gelidocalamus stellatus*

*Gelidocalamus subsolidus*

*Gelidocalamus tessellatus*

*Gelidocalamus velutinus*

*Gigantochloa achmadii*

*Gigantochloa albociliata*(4)*

*Gigantochloa albopilosa*

*Gigantochloa albovestita*

*Gigantochloa apus*(5)*

*Gigantochloa atroviolacea*(1)*

*Gigantochloa atter*(1)*

*Gigantochloa aya*

*Gigantochloa baliana*

*Gigantochloa balui*

*Gigantochloa bastareana*

*Gigantochloa calcicola*

*Gigantochloa cochinchinensis*

*Gigantochloa compressa*(1)*

*Gigantochloa densa*

*Gigantochloa dinhensis*

*Gigantochloa felix*

*Gigantochloa hasskarliana*(5)*

*Gigantochloa hayatae*

*Gigantochloa hirtinoda*

*Gigantochloa holttumiana*

*Gigantochloa hosseusii*

*Gigantochloa kuring*

*Gigantochloa latifolia*

*Gigantochloa levis*(2)*

*Gigantochloa ligulata*

*Gigantochloa longiprophylla*

*Gigantochloa luteostriata*(1)*

*Gigantochloa macrostachya*

*Gigantochloa magentea*

*Gigantochloa manggong*(1)*

*Gigantochloa membranoidea*

*Gigantochloa multiculmis*

*Gigantochloa multifloscula*

*Gigantochloa nigrociliata*

*Gigantochloa papyracea*

*Gigantochloa parvifolia*

*Gigantochloa poilanei*

*Gigantochloa pruriens*

*Gigantochloa pubinervis*

*Gigantochloa pubipetiolata*

*Gigantochloa ridleyi*(1)*

*Gigantochloa robusta*(1)*

*Gigantochloa rostrata*

*Gigantochloa scortechinii*

*Gigantochloa serik*

*Gigantochloa taluh*

*Gigantochloa tenuispiculata*

*Gigantochloa thoi*

*Gigantochloa tomentosa*

*Gigantochloa velutina*

*Gigantochloa verticillata*(6)*

*Gigantochloa vietnamica*

*Gigantochloa vinhphuica*

*Gigantochloa wallichiana*

*Gigantochloa wrayi*(1)*

*Glaziophyton mirabile*

*Greslania circinata*

*Greslania montana*

*Greslania multiflora*

*Greslania rivularis*

*Guadua aculeata*

*Guadua amplexifolia*(1)*

*Guadua angustifolia*(6)*

*Guadua calderoniana*

*Guadua chacoensis*

*Guadua chaparensis*

*Guadua ciliata*

*Guadua glomerata*

*Guadua incana*

*Guadua inermis*

*Guadua latifolia*

*Guadua lindmanii*

*Guadua longifolia*

*Guadua lynnclarkiae*

*Guadua macclurei*

*Guadua macrospiculata*

*Guadua macrostachya*

*Guadua maculosa*

*Guadua magna*

*Guadua paniculata*(1)*

*Guadua paraguayana*

*Guadua polyclados*

*Guadua refracta*(1)*

*Guadua sarcocarpa*

*Guadua superba*

*Guadua tagoara*

*Guadua takahashiae*

*Guadua trinii*(5)*

*Guadua uncinata*

*Guadua variegata*

*Guadua velutina*

*Guadua venezuelae*

*Guadua virgata*

*Guadua weberbaueri*

*Hickelia africana*

*Hickelia alaotrensis*

*Hickelia madagascariensis*

*Hickelia perrieri*(1)*

*Himalayacalamus asper*(1)*

*Himalayacalamus brevinodus*

*Himalayacalamus collaris*

*Himalayacalamus cupreus*

*Himalayacalamus falconeri*(6)*

*Himalayacalamus fimbriatus*

*Himalayacalamus hookerianus*(1)*

*Himalayacalamus planatus*

*Himalayacalamus porcatus*(1)*

*Hitchcockella baronii*

*Holttumochloa korbuensis*

*Holttumochloa magica*

*Holttumochloa pubescens*

*Indocalamus amplexicaulis*

*Indocalamus barbatus*

*Indocalamus bashanensis*

*Indocalamus chishuiensis*

*Indocalamus confertus*

*Indocalamus cordatus*

*Indocalamus decorus*

*Indocalamus emeiensis*

*Indocalamus guangdongensis*

*Indocalamus herklotsii*

*Indocalamus hirsutissimus*

*Indocalamus hirtivaginatus*

*Indocalamus hispidus*

*Indocalamus hunanensis*

*Indocalamus inaequilaterus*

*Indocalamus jinpingensis*

*Indocalamus latifolius*(1)*

*Indocalamus longiauritus*

*Indocalamus macrophyllus*

*Indocalamus multinervis*

*Indocalamus pedalis*

*Indocalamus petelotii*

*Indocalamus pseudosinicus*

*Indocalamus pumilus*

*Indocalamus quadratus*

*Indocalamus suichuanensis*

*Indocalamus tessellatus*(1)*

*Indocalamus tongchuensis*

*Indocalamus victorialis*

*Indocalamus youxiuensis*

*Indosasa angustata*

*Indosasa bacquangensis*

*Indosasa crassiflora*

*Indosasa gigantea*

*Indosasa glabrata*

*Indosasa hispida*

*Indosasa ingens*

*Indosasa laotica*

*Indosasa lipoensis*

*Indosasa longispicata*

*Indosasa lunata*

*Indosasa parvifolia*

*Indosasa patens*

*Indosasa shibataeaoides*

*Indosasa singulispicula*

*Indosasa sinica*(1)*

*Indosasa sondongensis*

*Indosasa spongiosa*

*Indosasa triangulata*

*Kinabaluchloa nebulosa*

*Kinabaluchloa wrayi*

*Kuruna debilis*

*Kuruna densifolia*

*Kuruna floribunda*

*Kuruna scandens*

*Kuruna walkeriana*

*Kuruna wightiana*

*Lithachne horizontalis*

*Lithachne humilis*

*Lithachne pauciflora*(1)*

*Lithachne pinetii*

*Maclurochloa montana*

*Maclurochloa tonkinensis*

*Maclurolyra tecta*

*Melocalamus arrectus*

*Melocalamus blaoensis*

*Melocalamus compactiflorus*

*Melocalamus cucphuongensis*

*Melocalamus elevatissimus*

*Melocalamus indicus*

*Melocalamus kbangensis*

*Melocalamus mastersii*

*Melocalamus ningmingensis*

*Melocalamus pacoensis*

*Melocalamus scandens*

*Melocalamus truongsonensis*

*Melocalamus yenbaiensis*

*Melocalamus yunnanensis*

*Melocanna baccifera*(4)*

*Melocanna humilis*

*Merostachys abadiana*

*Merostachys annulifera*

*Merostachys argentea*

*Merostachys argyronema*

*Merostachys bifurcata*

*Merostachys brevigluma*

*Merostachys brevispica*

*Merostachys burmanii*

*Merostachys calderoniana*

*Merostachys caucaiana*

*Merostachys ciliata*

*Merostachys claussenii*

*Merostachys exserta*

*Merostachys filgueirasii*

*Merostachys fimbriata*

*Merostachys fischeriana*

*Merostachys fistulosa*

*Merostachys glauca*

*Merostachys kleinii*

*Merostachys kunthii*

*Merostachys lanata*

*Merostachys latifolia*

*Merostachys leptophylla*

*Merostachys magellanica*

*Merostachys magnispicula*

*Merostachys maguireorum*

*Merostachys medullosa*

*Merostachys multiramea*

*Merostachys neesii*

*Merostachys pauciflora*

*Merostachys petiolata*

*Merostachys pilifera*

*Merostachys pluriflora*

*Merostachys polyantha*

*Merostachys procerrima*

*Merostachys ramosissima*

*Merostachys retrorsa*

*Merostachys riedeliana*

*Merostachys rondoniensis*

*Merostachys scandens*

*Merostachys sellovii*

*Merostachys skvortzovii*

*Merostachys sparsiflora*

*Merostachys speciosa*

*Merostachys tatianae*

*Merostachys ternata*

*Merostachys vestita*

*Merostachys yungasensis*

*Mniochloa pulchella*

*Mullerochloa moreheadiana*

*Myriocladus cardonae*

*Myriocladus churunensis*

*Myriocladus distantiflorus*

*Myriocladus exsertus*

*Myriocladus grandifolius*

*Myriocladus involutus*

*Myriocladus longiramosus*

*Myriocladus neblinaensis*

*Myriocladus paludicola*

*Myriocladus simplex*

*Myriocladus steyermarkii*

*Myriocladus virgatus*

*Nastus ambrensis*

*Nastus aristatus*

*Nastus borbonicus*

*Nastus decaryanus*

*Nastus elatoides*

*Nastus elatus*(1)*

*Nastus elegantissimus*

*Nastus elongatus*

*Nastus emirnensis*

*Nastus glaucus*

*Nastus holttumianus*

*Nastus hooglandii*

*Nastus humbertianus*

*Nastus humilus*

*Nastus lokohoensis*

*Nastus longispicula*

*Nastus madagascariensis*

*Nastus manongarivensis*

*Nastus obtusus*

*Nastus perrieri*

*Nastus productus*

*Nastus reholttumianus*

*Nastus rudimentifer*

*Nastus tsaratananensis*

*Neohouzeaua coradata*

*Neohouzeaua fimbriata*

*Neohouzeaua helferi*

*Neohouzeaua kerriana*

*Neohouzeaua mekongensis*(1)*

*Neohouzeaua puberula*

*Neohouzeaua stricta*

*Neohouzeaua tavoyana*

*Neololeba amahussana*

*Neololeba atra*

*Neololeba glabra*

*Neololeba hirsuta*

*Neololeba inaurita*

*Neomicrocalamus andropogonifolius*

*Neomicrocalamus clarkei*

*Neomicrocalamus dongvanensis*

*Neomicrocalamus prainii*

*Neomicrocalamus yunnanensis*

*Nianhochloa bidoupensis*

*Ochlandra beddomei*

*Ochlandra ebracteata*

*Ochlandra keralensis*

*Ochlandra scriptoria*

*Ochlandra setigera*

*Ochlandra sivagiriana*

*Ochlandra soderstromiana*

*Ochlandra spirostylis*

*Ochlandra stridula*

*Ochlandra talbotii*

*Ochlandra travancorica*

*Ochlandra wightii*

*Oldeania alpina*(1)*

*Oligostachyum bilobum*

*Oligostachyum exauriculatum*

*Oligostachyum glabrescens*

*Oligostachyum gracilipes*

*Oligostachyum hupehense*

*Oligostachyum lanceolatum*

*Oligostachyum lubricum*(1)*

*Oligostachyum nuspiculum*

*Oligostachyum oedogonatum*

*Oligostachyum paniculatum*

*Oligostachyum puberulum*

*Oligostachyum scabriflorum*

*Oligostachyum scopulum*

*Oligostachyum shiuyingianum*

*Oligostachyum spongiosum*

*Oligostachyum sulcatum*

*Oligostachyum wuyishanicum*

*Oligostachyum yongangense*

*Olmeca clarkiae*

*Olmeca fulgor*

*Olmeca recta*

*Olmeca reflexa*

*Olmeca zapotecorum*

*Olyra amapana*

*Olyra buchtienii*

*Olyra caudata*

*Olyra ciliatifolia*

*Olyra davidseana*

*Olyra ecaudata*

*Olyra fasciculata*

*Olyra filiformis*

*Olyra glaberrima*(1)*

*Olyra holttumiana*

*Olyra humilis*

*Olyra juruana*

*Olyra latifolia*?(2)*

*Olyra latispicula*

*Olyra longifolia*

*Olyra loretensis*

*Olyra maranonensis*

*Olyra obliquifolia*

*Olyra retrorsa*

*Olyra standleyi*(1)*

*Olyra tamanquareana*

*Olyra taquara*

*Olyra wurdackii*

*Oreobambos buchwaldii*

*Otatea acuminata*(2)*

*Otatea carrilloi*

*Otatea fimbriata*

*Otatea glauca*(1)*

*Otatea ramirezii*

*Otatea reynosoana*

*Otatea transvolcanica*

*Otatea ximenae*

*Oxytenanthera abyssinica*(6)*

*Parabambusa kainii*

*Pariana argentea*

*Pariana aurita*

*Pariana bicolor*

*Pariana campestris*

*Pariana carvalhoi*

*Pariana concinna*

*Pariana distans*

*Pariana ecuadorensis*

*Pariana gracilis*

*Pariana lanceolata*

*Pariana ligulata*

*Pariana maynensis*

*Pariana modesta*

*Pariana multiflora*

*Pariana nervata*

*Pariana obtusa*

*Pariana ovalifolia*

*Pariana pallida*

*Pariana parvispica*

*Pariana radiciflora*

*Pariana setosa*

*Pariana simulans*

*Pariana sociata*

*Pariana stenolemma*

*Pariana strigosa*

*Pariana swallenii*

*Pariana tenuis*

*Pariana trichosticha*

*Pariana ulei*

*Pariana velutina*

*Parodiolyra aratitiyopensis*

*Parodiolyra colombiensis*

*Parodiolyra lateralis*

*Parodiolyra luetzelburgii*

*Parodiolyra micrantha*(1)*

*Parodiolyra ramosissima*

*Perrierbambus madagascariensis*

*Perrierbambus tsarasaotrensis*

*Phuphanochloa speciosa*

*Phyllosasa tranquillans*

*Phyllostachys acuta*(4)*

*Phyllostachys acutiligula*

*Phyllostachys angusta*(1)*

*Phyllostachys arcana*(4)*

*Phyllostachys atrovaginata*(2)*

*Phyllostachys aurea*†(45)*

*Phyllostachys aureosulcata*(6)*

*Phyllostachys bambusoides*†(6)*

*Phyllostachys bissetii*(5)*

*Phyllostachys carnea*

*Phyllostachys circumpilis*(1)*

*Phyllostachys compressus*

*Phyllostachys corrugata*

*Phyllostachys dulcis*(4)*

*Phyllostachys edulis*†(15)*

*Phyllostachys elegans*

*Phyllostachys fimbriligula*

*Phyllostachys flexuosa*†(9)*

*Phyllostachys glabrata*(1)*

*Phyllostachys glauca*(4)*

*Phyllostachys guizhouensis*

*Phyllostachys heteroclada*(2)*

*Phyllostachys hirtivagina*

*Phyllostachys incarnata*(1)*

*Phyllostachys iridescens*

*Phyllostachys kwangsiensis*

*Phyllostachys lofushanensis*(1)*

*Phyllostachys makinoi*(1)*

*Phyllostachys mannii*(4)*

*Phyllostachys meyeri*(5)*

*Phyllostachys mirabilis*

*Phyllostachys nidularia*(7)*

*Phyllostachys nigella*(1)*

*Phyllostachys nigra*†(26)*

*Phyllostachys nuda*(7)*

*Phyllostachys parvifolia*

*Phyllostachys pierreana*

*Phyllostachys platyglossa*

*Phyllostachys prominens*

*Phyllostachys propinqua*(1)*

*Phyllostachys purpureociliata*

*Phyllostachys reticulata*

*Phyllostachys rivalis*(2)*

*Phyllostachys robustiramea*(1)*

*Phyllostachys rubicunda*

*Phyllostachys rubromarginata*(4)*

*Phyllostachys rutila*

*Phyllostachys shuchengensis*

*Phyllostachys stimulosa*(3)*

*Phyllostachys sulphurea*(6)*

*Phyllostachys tianmuensis*

*Phyllostachys varioauriculata*

*Phyllostachys veitchiana*

*Phyllostachys verrucosa*

*Phyllostachys violascens*(5)*

*Phyllostachys virella*

*Phyllostachys viridiglaucescens*(7)*

*Phyllostachys vivax*(6)*

*Phyllostachys yunhoensis*

*Pinga marginata*

*Piresia goeldii*

*Piresia leptophylla*

*Piresia macrophylla*

*Piresia palmula*

*Piresia sympodica*

*Piresiella strephioides*

*Pleioblastus albosericeus*

*Pleioblastus altiligulatus*

*Pleioblastus amarus*

*Pleioblastus argenteostriatus*†(22)*

*Pleioblastus chino*(1)*

*Pseudosasa distichus*†(4)*

*Pleioblastus gozadakensis*

*Pleioblastus gramineus*(6)*

*Pleioblastus guilongshanensis*

*Pleioblastus hattorianus*

*Pleioblastus hsienchuensis*

*Pseudosasa humilis*†(8)*

*Pleioblastus incarnatus*

*Pleioblastus intermedius*

*Pleioblastus juxianensis*

*Pleioblastus kodzumae*

*Pleioblastus kongosanensis*

*Pleioblastus linearis*(3)*

*Pleioblastus maculatus*

*Pleioblastus maculosoides*

*Pleioblastus matsunoi*

*Pleioblastus nagashima*(3)*

*Pleioblastus oleosus*

*Pleioblastus patellaris*

*Pleioblastus pseudocommunis*

*Pleioblastus pseudosasaoides*

*Pleioblastus rugatus*

*Pleioblastus sanmingensis*

*Pleioblastus simonii*?(15)*

*Pleioblastus solidus*

*Pleioblastus truncatus*

*Pleioblastus variegatus*(12)*

*Pleioblastus wuyishanensis*(1)*

*Pleioblastus yamadorianus*

*Pleioblastus yixingensis*

*Pseudobambusa schizostachyoides*

*Pseudosasa aeria*

*Pseudosasa amabilis*(2)*

*Pseudosasa amplexicaulis*

*Pseudosasa brevivaginata*

*Pseudosasa cantorii*(1)*

*Pseudosasa gracilis*

*Pseudosasa hindsii*(8)*

*Pseudosasa japonica*?(23)*

*Pseudosasa jiangleensis*

*Pseudosasa longiligula*

*Pseudosasa maculifera*

*Pseudosasa membraniligulata*

*Pseudosasa nabeshimana*

*Pseudosasa orthotropa*

*Pseudosasa owatarii*

*Pseudosasa pubiflora*

*Pseudosasa subsolida*

*Pseudosasa viridula*

*Pseudosasa wuyiensis*

*Pseudosasa zhongyanensis*

*Pseudostachyum polymorphum*(1)*

*Pseudostachyum wakha*

*Pseudoxytenanthera bourdillonii*

*Pseudoxytenanthera monadelpha*

*Pseudoxytenanthera parvifolia*

*Pseudoxytenanthera ritcheyi*

*Pseudoxytenanthera stocksii*

*Racemobambos celebica*

*Racemobambos ceramica*

*Racemobambos congesta*

*Racemobambos gibbsiae*

*Racemobambos glabra*

*Racemobambos hepburnii*

*Racemobambos hirsuta*

*Racemobambos hirta*

*Racemobambos holttumii*

*Racemobambos kutaiensis*

*Racemobambos multiramosa*

*Racemobambos novohibernica*

*Racemobambos pairinii*

*Racemobambos raynalii*

*Racemobambos rigidifolia*

*Racemobambos rupicola*

*Racemobambos schultzei*

*Racemobambos sessilis*

*Racemobambos setifera*

*Raddia angustifolia*

*Raddia brasiliensis*

*Raddia distichophylla*

*Raddia guianensis*

*Raddia lancifolia*

*Raddia megaphylla*

*Raddia portoi*

*Raddia soderstromii*

*Raddia stolonifera*

*Raddiella esenbeckii*(1)*

*Raddiella kaieteurana*

*Raddiella lunata*

*Raddiella malmeana*

*Raddiella minima*

*Raddiella molliculma*

*Raddiella potaroensis*

*Raddiella vanessiae*

*Rehia nervata*

*Reitzia smithii*

*Rhipidocladum abregoense*

*Rhipidocladum ampliflorum*

*Rhipidocladum angustiflorum*

*Rhipidocladum arenicola*

*Rhipidocladum bartlettii*

*Rhipidocladum clarkiae*

*Rhipidocladum cordatum*

*Rhipidocladum harmonicum*

*Rhipidocladum martinezii*

*Rhipidocladum maxonii*

*Rhipidocladum neumannii*

*Rhipidocladum pacuarense*

*Rhipidocladum panamense*

*Rhipidocladum parviflorum*

*Rhipidocladum pittieri*

*Rhipidocladum prestoei*

*Rhipidocladum racemiflorum*

*Rhipidocladum rubrofimbriatum*

*Rhipidocladum sibilans*

*Sarocalamus faberi*

*Sarocalamus racemosus*

*Sarocalamus spanostachyus*

*Sasa bitchuensis*(1)*

*Sasa cernua*

*Sasa chartacea*

*Sasa elegantissima*(2)*

*Sasa fugeshiensis*

*Sasa gracillima*

*Sasa guangdongensis*

*Sasa guangxiensis*

*Sasa hainanensis*

*Sasa hayatae*

*Sasa heterotricha*

*Sasa hibaconuca*

*Sasa hidaensis*

*Sasa hisauchii*

*Sasa kagamiana*

*Sasa kanayamensis*

*Sasa kogasensis*

*Sasa kurilensis*(1)*

*Sasa kurokawana*

*Sasa longiligulata*

*Sasa magnifica*

*Sasa magnonoda*

*Sasa masamuneana*(3)*

*Sasa megalogluma*

*Sasa megalophylla*

*Sasa miakeana*

*Sasa minensis*

*Sasa nipponica*(1)*

*Sasa oblongula*

*Sasa occidentalis*

*Sasa oshidensis*

*Sasa palmata*(13)*

*Sasa pubens*

*Sasa pubiculmis*

*Sasa pulcherrima*

*Sasa quelpaertensis*(1)*

*Sasa ramosa*(6)*

*Sasa rubrovaginata*

*Sasa sadoensis*

*Sasa samaniana*

*Sasa scytophylla*

*Sasa senanensis*(2)*

*Sasa septentrionalis*

*Sasa shimidzuana*

*Sasa sirakurensis*

*Sasa subglabra*

*Sasa subvillosa*

*Sasa suzukii*

*Sasa takizawana*

*Sasa tatewakiana*

*Sasa tenuifolia*

*Sasa tokugawana*

*Sasa tomentosa*

*Sasa tsuboiana*(2)*

*Sasa tsukubensis*

*Sasa veitchii*(9)*

*Sasa yahikoensis*

*Sasaella bitchuensis*

*Sasaella caudiceps*

*Sasaella hidaensis*

*Sasaella hisauchii*

*Sasaella iwatekensis*

*Sasaella kogasensis*

*Sasaella leucorhoda*

*Sasaella masamuneana*

*Sasaella ovarifolia*

*Sasaella ramosa*

*Sasaella sadoensis*

*Sasaella shiobarensis*

*Sasamorpha borealis*(2)*

*Sasamorpha hubeiensis*

*Sasamorpha oshidensis*

*Sasamorpha qingyuanensis*

*Sasamorpha sinica*(1)*

*Schizostachyum aciculare*

*Schizostachyum aequiramosum*

*Schizostachyum alopecurus*

*Schizostachyum andamanicum*

*Schizostachyum atrocingulare*

*Schizostachyum auriculatum*

*Schizostachyum bamban*

*Schizostachyum blumei*

*Schizostachyum brachycladum*(2)*

*Schizostachyum brachythyrsus*

*Schizostachyum castaneum*

*Schizostachyum caudatum*

*Schizostachyum chinense*

*Schizostachyum copelandii*

*Schizostachyum cornutum*

*Schizostachyum curranii*

*Schizostachyum cuspidatum*

*Schizostachyum diaoluoshanense*

*Schizostachyum distans*

*Schizostachyum dullooa*

*Schizostachyum dumetorum*(2)*

*Schizostachyum flexuosum*

*Schizostachyum funghomii*(1)*

*Schizostachyum glaucifolium*(3)*

*Schizostachyum glaucocladum*

*Schizostachyum gracile*

*Schizostachyum grande*

*Schizostachyum hainanense*

*Schizostachyum hantu*

*Schizostachyum insulare*

*Schizostachyum iraten*

*Schizostachyum jaculans*(2)*

*Schizostachyum kalpongianum*

*Schizostachyum khoonmengii*

*Schizostachyum latifolium*

*Schizostachyum lengguanii*

*Schizostachyum lima*(1)*

*Schizostachyum lumampao*(2)*

*Schizostachyum lutescens*

*Schizostachyum mampouw*

*Schizostachyum mannii*

*Schizostachyum nghianum*

*Schizostachyum ninhthuanense*

*Schizostachyum perrieri*

*Schizostachyum pilosum*

*Schizostachyum pingbianense*

*Schizostachyum pleianthemum*

*Schizostachyum pseudolima*(1)*

*Schizostachyum rogersii*

*Schizostachyum sanguineum*

*Schizostachyum seshagirianum*

*Schizostachyum silicatum*

*Schizostachyum terminale*

*Schizostachyum tessellatum*

*Schizostachyum textorium*

*Schizostachyum undulatum*

*Schizostachyum wanshishanense*

*Schizostachyum whitei*

*Schizostachyum yalyense*

*Schizostachyum zollingeri*(1)*

*Shibataea chiangshanensis*

*Shibataea chinensis*(2)*

*Shibataea hispida*(1)*

*Shibataea kumasasa*(9)*

*Shibataea lancifolia*(1)*

*Shibataea nanpingensis*

*Shibataea strigosa*

*Sinobambusa baccanensis*

*Sinobambusa farinosa*

*Sinobambusa glabrata*

*Sinobambusa henryi*

*Sinobambusa humila*

*Sinobambusa incana*

*Sinobambusa intermedia*(2)*

*Sinobambusa nephroaurita*

*Sinobambusa rubroligula*(3)*

*Sinobambusa sat*

*Sinobambusa seminuda*

*Sinobambusa solearis*

*Sinobambusa tootsik*(6)*

*Sinobambusa yixingensis*

*Sirochloa parvifolia*

*Soejatmia ridleyi*

*Sphaerobambos hirsuta*

*Sphaerobambos philippinensis*

*Sphaerobambos subtilis*

*Stapletonia arunachalensis*

*Sucrea maculata*

*Sucrea monophylla*

*Sucrea sampaiana*

*Teinostachyum beddomei*

*Teinostachyum griffithii*

*Temburongia simplex*

*Temochloa liliana*

*Thamnocalamus chigar*

*Thamnocalamus spathiflorus*(5)*

*Thamnocalamus unispiculatus*

*Thyrsostachys oliveri*(1)*

*Thyrsostachys siamensis*(3)*

*Valiha diffusa*

*Valiha perrieri*

*Vietnamocalamus catbaensis*

*Vietnamosasa ciliata*

*Vietnamosasa darlacensis*

*Vietnamosasa pusilla*

*Yushania addingtonii*

*Yushania ailuropodina*

*Yushania anceps*(7)*

*Yushania andropogonoides*

*Yushania angustifolia*

*Yushania auctiaurita*

*Yushania baishanzuensis*

*Yushania basihirsuta*

*Yushania bojieiana*

*Yushania brevipaniculata*(1)*

*Yushania brevis*

*Yushania burmanica*

*Yushania canoviridis*

*Yushania cartilaginea*

*Yushania cava*

*Yushania chingii*

*Yushania collina*

*Yushania complanata*

*Yushania confusa*

*Yushania crassicollis*

*Yushania crispata*

*Yushania dafengdingensis*

*Yushania donganensis*

*Yushania elegans*

*Yushania elevata*

*Yushania exilis*

*Yushania falcatiaurita*

*Yushania farcticaulis*

*Yushania farinosa*

*Yushania flexa*

*Yushania glandulosa*

*Yushania glauca*

*Yushania grammata*

*Yushania hirsuta*

*Yushania hirticaulis*

*Yushania humbertii*

*Yushania lacera*

*Yushania laetevirens*

*Yushania levigata*

*Yushania linearis*

*Yushania lineolata*

*Yushania longiaurita*

*Yushania longissima*(6)*

*Yushania longiuscula*

*Yushania mabianensis*

*Yushania maculata*

*Yushania madagascariensis*

*Yushania maling*(1)*

*Yushania menghaiensis*

*Yushania microphylla*

*Yushania mitis*

*Yushania multiramea*

*Yushania niitakayamensis*(4)*

*Yushania oblonga*

*Yushania pachyclada*

*Yushania pantlingii*

*Yushania pauciramificans*

*Yushania perrieri*

*Yushania polytricha*

*Yushania punctulata*

*Yushania qiaojiaensis*

*Yushania rigidula*

*Yushania rolloana*

*Yushania rugosa*

*Yushania shangrilaensis*

*Yushania straminea*

*Yushania suijiangensis*

*Yushania tenuicaulis*

*Yushania tessellata*

*Yushania uniramosa*

*Yushania varians*

*Yushania velutina*

*Yushania vigens*

*Yushania violascens*

*Yushania wardii*

*Yushania weixiensis*

*Yushania wuyishanensis*

*Yushania xizangensis*

*Yushania yadongensis*

*Yushania yongdeensis*

**References**

Kellogg EA. 2015. Poaceae. In: Kubitzki K. (Ed.) The families and genera of vascular plants. Flowering Plants. Monocots: Poaceae (Vol. 13). Springer, Cham.

**Table S2.** List of named bamboo species that did not match our accepted species list and the changes made to include or exclude from the review database. Synonyms and spelling mistakes were updated accordingly, and unknown names were excluded.

| **Name used in records** | **Updated name** | **Issue** |
| --- | --- | --- |
| *Arundinaria amabilis* | *Pseudosasa amabilis* | Synonym |
| *Arundinaria debilis* | *Kuruna debilis* | Synonym |
| *Arundinaria densifolia* | *Kuruna densifolia* | Synonym |
| *Arundinaria faberi* | *Sarocalamus faberi* | Synonym |
| *Arundinaria fargesii* | *Bashania fargesii* | Synonym |
| *Arundinaria floribunda* | *Kuruna floribunda* | Synonym |
| *Arundinaria funghomii* | *Pseudosasa cantorii* | Synonym |
| *Arundinaria graminea* | *Pleioblastus gramineus* | Synonym |
| *Arundinaria hindsii* | *Pseudosasa hindsii* | Synonym |
| *Arundinaria hookeriana* | *Himalayacalamus hookerianus* | Synonym |
| *Arundinaria humilis* | *Pseudosasa humilis* | Synonym |
| *Arundinaria japonica* | *Pseudosasa japonica* | Synonym |
| *Arundinaria kodzumae* | *Pleioblastus kodzumae* | Synonym |
| *Arundinaria kongosanensis* | *Pleioblastus kongosanensis* | Synonym |
| *Arundinaria linearis* | *Pleioblastus linearis* | Synonym |
| *Arundinaria matsunoi* | *Pleioblastus matsunoi* | Synonym |
| *Arundinaria nagashima* | *Pleioblastus nagashima* | Synonym |
| *Arundinaria nitida* | *Fargesia nitida* | Synonym |
| *Arundinaria pygmaea* | *Pleioblastus variegatus* | Synonym |
| *Arundinaria qingchengshanensis* | *Bashania qingchengshanensis* | Synonym |
| *Arundinaria racemosa* | *Sarocalamus racemosus* | Synonym |
| *Arundinaria scandens* | *Kuruna scandens* | Synonym |
| *Arundinaria shiobarensis* | *Sasaella shiobarensis* | Synonym |
| *Arundinaria simonii* | *Pleioblastus simonii* | Synonym |
| *Arundinaria spanostachya* | *Sarocalamus spanostachyus* | Synonym |
| *Arundinaria usawai* | *Pseudosasa japonica* | Synonym |
| *Arundinaria variegata* | *Pleioblastus variegatus* | Synonym |
| *Arundinaria viridistriata* | *Pleioblastus variegatus* | Synonym |
| *Arundinaria viridi-striata* | *Pleioblastus variegatus* | Synonym |
| *Arundinaria walkeriana* | *Kuruna walkeriana* | Synonym |
| *Arundinaria wightiana* | *Kuruna wightiana* | Synonym |
| *Aulonemia clarkiae* | *Olmeca clarkiae* | Synonym |
| *Aulonemia fulgor* | *Olmeca fulgor* | Synonym |
| *Bambusa amplexifolia* | *Guadua amplexifolia* | Synonym |
| *Bambusa argenteostriata* | *Pleioblastus argenteostriatus* | Synonym |
| *Bambusa arundinacea* | *Bambusa bambos* | Synonym |
| *Bambusa aurea* | *Phyllostachys aurea* | Synonym |
| *Bambusa castilloni* | *Phyllostachys bambusoides* | Synonym |
| *Bambusa glaucescens* | *Bambusa multiplex* | Synonym |
| *Bambusa guadua* | *Guadua angustifolia* | Synonym |
| *Bambusa horsfieldii* | *Fimbribambusa horsfieldii* | Synonym |
| *Bambusa microcephala* | *Fimbribambusa microcephala* | Synonym |
| *Bambusa moreheadiana* | *Mullerochloa moreheadiana* | Synonym |
| *Bambusa nana* | *Bambusa multiplex* | Synonym |
| *Bambusa paniculata* | *Sasa senanensis* | Synonym |
| *Bambusa pygmaea* | *Pleioblastus variegatus* | Synonym |
| *Bambusa shimadai* | *Bambusa multiplex* | Synonym |
| *Bambusa ventricosa* | *Bambusa tuldoides* | Synonym |
| *Chimonobambusa falcata* | *Drepanostachyum falcatum* | Synonym |
| *Dendrocalamus beecheyana* | *Bambusa beecheyana* | Synonym |
| *Dendrocalamus latifolius* | *Dendrocalamus latiflorus* | Synonym |
| *Drepanostachyum asper* | *Himalayacalamus asper* | Synonym |
| **Name used in records** | **Updated name** | **Issue** |
| *Drepanostachyum porcatum* | *Himalayacalamus porcatus* | Synonym |
| *Eremocaulon setosum* | *Aulonemia setosa* | Synonym |
| *Gigantochloa parviflora* | *Gigantochloa parvifolia* | Synonym |
| *Gigantochloa pseudoarundinacea* | *Gigantochloa verticillata* | Synonym |
| *Greslania circinnata* | *Greslania circinata* | Synonym |
| *Indocalamus wilsonii* | *Indocalamus wilsonii* | Synonym |
| *Leleba oldhami* | *Bambusa oldhamii* | Synonym |
| *Leleba shimadai* | *Bambusa multiplex* | Synonym |
| *Melocanna arundina* | *Melocanna humilis* | Synonym |
| *Melocanna virgata* | *Cephalostachyum virgatum* | Synonym |
| *Melocanna zollingeri* | *Schizostachyum zollingeri* | Synonym |
| *Mniochloa strephioides* | *Piresiella strephioides* | Synonym |
| *Neurolepis angusta* | *Chusquea angusta* | Synonym |
| *Neurolepis aperta* | *Chusquea spectabilis* | Synonym |
| *Neurolepis aristata* | *Chusquea aristata* | Synonym |
| *Neurolepis asymmetrica* | *Chusquea asymmetrica* | Synonym |
| *Neurolepis diversiglumis* | *Chusquea diversiglumis* | Synonym |
| *Neurolepis elata* | *Chusquea elata* | Synonym |
| *Neurolepis fimbriligulata* | *Chusquea fimbriligulata* | Synonym |
| *Neurolepis glomerata* | *Chusquea glomerata* | Synonym |
| *Neurolepis laegaardii* | *Chusquea laegaardii* | Synonym |
| *Neurolepis mollis* | *Chusquea mollis* | Synonym |
| *Neurolepis nana* | *Chusquea nana* | Synonym |
| *Neurolepis petiolata* | *Chusquea petiolata* | Synonym |
| *Neurolepis pittieri* | *Chusquea pittieri* | Synonym |
| *Neurolepis rigida* | *Chusquea rigida* | Synonym |
| *Neurolepis silverstonei* | *Chusquea silverstonei* | Synonym |
| *Neurolepis villosa* | *Chusquea villosa* | Synonym |
| *Neurolepis virgata* | *Chusquea virgata* | Synonym |
| *Olyra bahiensis* | *Arberella bahiensis* | Synonym |
| *Olyra micrantha* | *Parodiolyra micrantha* | Synonym |
| *Otatea aztecorum* | *Otatea acuminata* | Synonym |
| *Pariana gleasonii* | *Pariana radiciflora* | Synonym |
| *Pariana intermedia* | *Pariana campestris* | Synonym |
| *Pariana interrupta* | *Pariana campestris* | Synonym |
| *Pariana longiflora* | *Pariana radiciflora* | Synonym |
| *Pariana lunata* | *Pariana campestris* | Synonym |
| *Pariana vulgaris* | *Pariana radiciflora* | Synonym |
| *Phyllostachys heterocycla* | *Phyllostachys edulis* | Synonym |
| *Phyllostachys praecox* | *Phyllostachys violascens* | Synonym |
| *Phyllostachys pubescens* | *Phyllostachys edulis* | Synonym |
| *Phyllostachys quilioi* | *Phyllostachys bambusoides* | Synonym |
| *Phyllostachys viridis* | *Phyllostachys sulphurea* | Synonym |
| *Pleioblastus fortunei* | *Pleioblastus variegatus* | Synonym |
| *Pleioblastus hindsii* | *Pseudosasa hindsii* | Synonym |
| *Pleioblastus humilis* | *Pseudosasa humilis* | Synonym |
| *Pleioblastus pygmaeus* | *Pleioblastus variegatus* | Synonym |
| *Pleioblastus shibuyanus* | *Pleioblastus variegatus* | Synonym |
| *Pleioblastus tsukubensis* | *Sasa tsukubensis* | Synonym |
| *Pleioblastus viridistriatus* | *Bambusa viridis* | Synonym |
| *Pseudosasa disticha* | *Pseudosasa distichus* | Synonym |
| *Pseudosasa usawai* | *Pseudosasa japonica* | Synonym |
| **Name used in records** | **Updated name** | **Issue** |
| *Pseudoxytenanthera albociliata* | *Gigantochloa albociliata* | Synonym |
| *Rhipidocladum geminatum* | *Didymogonyx geminatum* | Synonym |
| *Rhipidocladum longispiculatum* | *Didymogonyx longispiculatum* | Synonym |
| *Sasa admirabilis* | *Sasa elegantissima* | Synonym |
| *Sasa argenteostriata* | *Pleioblastus argenteostriatus* | Synonym |
| *Sasa borealis* | *Sasamorpha borealis* | Synonym |
| *Sasa fortunei* | *Pleioblastus variegatus* | Synonym |
| *Sasa hubeiensis* | *Sasamorpha hubeiensis* | Synonym |
| *Sasa qingyuanensis* | *Sasamorpha qingyuanensis* | Synonym |
| *Sasa sinica* | *Sasamorpha sinica* | Synonym |
| *Sasa tessellata* | *Indocalamus tessellatus* | Synonym |
| *Sasaella glabra* | *Sasa masamuneana* | Synonym |
| *Schizostachyum arunachalensis* | *Stapletonia arunachalensis* | Synonym |
| *Schizostachyum beddomei* | *Teinostachyum beddomei* | Synonym |
| *Schizostachyum diffusum* | *Dinochloa diffusa* | Synonym |
| *Schizostachyum griffithii* | *Teinostachyum griffithii* | Synonym |
| *Schizostachyum polymorphum* | *Pseudostachyum polymorphum* | Synonym |
| *Semiarundinaria densiflora* | *Semiarundinaria densiflora* | Synonym |
| *Semiarundinaria fastuosa* | *Semiarundinaria fastuosa* | Synonym |
| *Semiarundinaria fortis* | *Semiarundinaria fortis* | Synonym |
| *Semiarundinaria kagamiana* | *Semiarundinaria kagamiana* | Synonym |
| *Semiarundinaria shapoensis* | *Semiarundinaria shapoensis* | Synonym |
| *Semiarundinaria sinica* | *Semiarundinaria sinica* | Synonym |
| *Semiarundinaria yashadake* | *Semiarundinaria yashadake* | Synonym |
| *Shibataea lanceifolia* | *Shibataea lancifolia* | Synonym |
| *Sinobambusa kunishii* | *Gelidocalamus kunishii* | Synonym |
| *Sinocalamus latiflorus* | *Dendrocalamus latiflorus* | Synonym |
| *Thamnocalamus falconeri* | *Himalayacalamus falconeri* | Synonym |
| *Thamnocalamus nitidus* | *Fargesia nitida* | Synonym |
| *Thamnocalamus spathaceus* | *Fargesia spathacea* | Synonym |
| *Thamnocalamus tessellatus* | *Bergbambos tessellata* | Synonym |
| *Yushania alpina* | *Oldeania alpina* | Synonym |
| *Arundinaria caudiceps* | *Sasaella caudiceps* | Synonym |
| *Arundinaria chino* | *Pleioblastus argenteostriatus* | Synonym |
| *Gigantochloa parviflora* | *Gigantochloa parvifolia* | Spelling |
| *Greslania circinnata* | *Greslania circinata* | Spelling |
| *Guadua angus* | *Guadua angustifolia* | Spelling |
| *Indocalamus wilsonii* | *Indocalamus wilsonii* | Spelling |
| *Merostachys cauciana* | *Merostachys caucaiana* | Spelling |
| *Pariana ecuadoriensis* | *Pariana ecuadorensis* | Spelling |
| *Parodiolyra columbiensis* | *Parodiolyra colombiensis* | Spelling |
| *Pleioblastus gramineaus* | *Pleioblastus gramineus* | Spelling |
| *Pleioblastus humili* | *Pleioblastus humilis* | Spelling |
| *Pleioblastus viridistriatus* | *Bambusa viridis* | Spelling |
| *Rhipidocladum abregoensis* | *Rhipidocladum abregoense* | Spelling |
| *Schizostachyum blumii* | *Schizostachyum blumei* | Spelling |
| *Shibataea kumasaca* | *Shibataea kumasasa* | Spelling |
| *Shibataea lanceifolia* | *Shibataea lancifolia* | Spelling |
| *Yushania grummata* | *Yushania grammata* | Spelling |
| *Yushania suijangensis* | *Yushania suijiangensis* | Spelling |
| *Arthrostylidium multispicatum* |  | Unknown name/ excluded |
| *Arundinaria auricom* |  | Unknown name/ excluded |
| *Arundinaria fansipanensis* |  | Unknown name/ excluded |
| *Arundinaria munsuensis* |  | Unknown name/ excluded |
| *Arundinaria pseudosasaoides* |  | Unknown name/ excluded |
| **Name used in records** | **Updated name** | **Issue** |
| *Aulonemia sodiroana* |  | Unknown name/ excluded |
| *Aulonemia ulei* |  | Unknown name/ excluded |
| *Bambusa schizostachyoides* |  | Unknown name/ excluded |
| *Bonia saxatilis* |  | Unknown name/ excluded |
| *Drepanostachyum exauritum* |  | Unknown name/ excluded |
| *Fargesia chigar* |  | Unknown name/ excluded |
| *Indocalamus sinicus* |  | Unknown name/ excluded |
| *Kinabaluchloa ridleyi* |  | Unknown name/ excluded |
| *Myriocladus maguirei* |  | Unknown name/ excluded |
| *Nastus schlechteri* |  | Unknown name/ excluded |
| *Nastus schmutzii* |  | Unknown name/ excluded |
| *Phyllostachys humilis* |  | Unknown name/ excluded |
| *Phyllostachys octandra* |  | Unknown name/ excluded |
| *Pleioblastus auricomus* |  | Unknown name/ excluded |
| *Pseudosasa hamadae* |  | Unknown name/ excluded |
| *Sasa mollissima* |  | Unknown name/ excluded |
| *Schizostachyum biflorum* |  | Unknown name/ excluded |
| *Sinoarundinaria falcata* |  | Unknown name/ excluded |
| *Sinocalamus oldhammi* |  | Unknown name/ excluded |

**
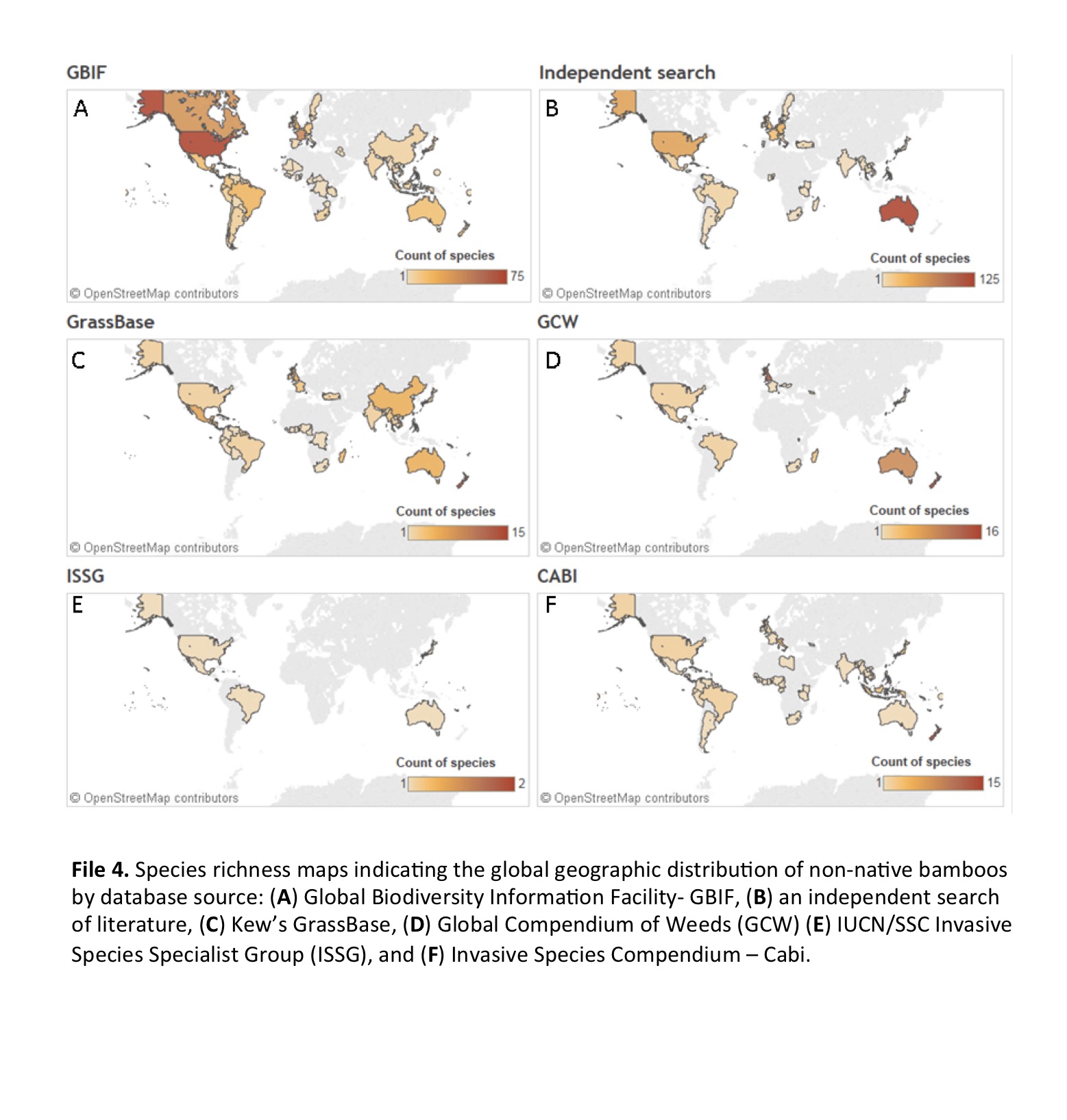
**

**Figure S1.** Species richness maps indicating the global geographic distribution of non-native bamboos by database source: (A) Global Biodiversity Information Facility- GBIF, (B) an independent search of literature, (C) Kew’s GrassBase, (D) Global Compendium of Weeds (GCW) (E) IUCN/SSC Invasive Species Specialist Group (ISSG), and (F) Invasive Species Compendium – CABI.

Table S3. List of references for bamboo invasions and the locality (‘Country/ Region’) of the reported invasion. The ‘Database/ Report’ indicates where references were originally retrieved. All references were vetted for validity on invasion claim (see Richardson et al. 2011), ‘Reference Status’ indicates which references were included or excluded in the global review.

| **Species** | **Country/ Region** | **Database/ Report** | **Reference Status** | **Reference** |
| --- | --- | --- | --- | --- |
| *Phyllostachys aurea* | Australia | Pacific Island Ecosystems at Risk (PIER) | Included | (Queensland Herbarium 2002) |
| *Phyllostachys aurea* | Brazil | A Global Compendium of Weeds (Randall, 2012) | Included | (IABIN2012) |
| *Bambusa textilis* | Brazil | A Global Compendium of Weeds (Randall, 2012) | Included | (IABIN 2008) |
| *Bambusa vulgaris* | Brazil | A Global Compendium of Weeds (Randall, 2012) | Included | (IABIN 2012) |
| *Bambusa bambos* | Cuba | Invasive Species Compendium (CABI) | Included | (Oviendo 2012) |
| *Bambusa vulgaris* | Cuba | Invasive Species Compendium (CABI) | Included | (Oviendo 2012) |
| *Dendrocalamus strictus* | Cuba | Invasive Species Compendium (CABI) | Included | (Oviendo 2012) |
| *Phyllostachys aurea* | Cuba | Invasive Species Compendium (CABI) | Included | (Oviendo 2012) |
| *Phyllostachys bambusoides* | Cuba | Invasive Species Compendium (CABI) | Included | (Oviendo 2012) |
| *Phyllostachys flexuosa* | Cuba | Invasive Species Compendium (CABI) | Included | (Oviendo 2012) |
| *Pleioblastus argenteostriatus* | Georgia | A Global Compendium of Weeds (Randall, 2012) | Included | (Kikodze 2010) |
| *Pseudosasa distichus* | Georgia | A Global Compendium of Weeds (Randall, 2012) | Included | (Kikodze 2010) |
| *Pseudosasa humilis* | Georgia | A Global Compendium of Weeds (Randall, 2012) | Included | (Kikodze 2010) |
| *Phyllostachys aurea* | Hawaii | Pacific Island Ecosystems at Risk (PIER) | Included | (Staples et al. 2002) |
| *Phyllostachys nigra* | Hawaii | Pacific Island Ecosystems at Risk (PIER) | Included | (Wagner et al. 1999; Tunison et al. 2000) |
| *Bambusa vulgaris* | Jamaica | A Global Compendium of Weeds (Randall, 2012) | Included | (Kairo et al. 2003) |
| *Phyllostachys edulis* | Japan | A Global Compendium of Weeds (Randall, 2012) | Included | (Toshiya 2004) |
| *Phyllostachys aurea* | New Zealand | Pacific Island Ecosystems at Risk (PIER) | Included | (Edgar & Connor, 2000) |
| *Bambusa vulgaris* | Puerto Rico | Invasive Species Compendium (CABI) | Included | (Blundell et al. 2003) |
| *Dendrocalamus strictus* | Puerto Rico | A Global Compendium of Weeds (Randall, 2012) | Included | (Kairo et al. 2003) |
| *Bambusa vulgaris* | Trinidad and Tobago | A Global Compendium of Weeds (Randall, 2012) | Included | (Kairo et al. 2003) |
| *Phyllostachys aurea* | United States | A Global Compendium of Weeds (Randall, 2012) | Included | (Bargeron 2003; Swearingen et al. 2010; Gucker 2009) |
| *Bambusa balcooa* | Australia | A Global Compendium of Weeds (Randall, 2012) | Not Accessible | (Keighery & Keighery 2009) |
| *Bambusa vulgaris* | Costa Rica | Invasive Species Compendium (CABI) | Not Accessible | (Chacón & Saborío, 2012) |
| *Bambusa vulgaris* | Galapagos Is. | Invasive Species Compendium (CABI) | Not Accessible | (Charles Darwin Foundation 2014) |
| *Phyllostachys aurea* | Japan | A Global Compendium of Weeds (Randall, 2012) | Not Accessible | (Toshiya 2004) |
| *Phyllostachys nigra* | Japan | A Global Compendium of Weeds (Randall, 2012) | Not Accessible | (Toshiya 2004) |
| *Bambusa multiplex* | Reunion Is. | Pacific Island Ecosystems at Risk (PIER) | Not included | (Lavergne 2006) |
| *Bambusa vulgaris* | Reunion Is. | Pacific Island Ecosystems at Risk (PIER) | Not included | (Lavergne 2006) |
| *Phyllostachys aurea* | Reunion Is. | Pacific Island Ecosystems at Risk (PIER) | Not included | (Lavergne 2006) |
| *Phyllostachys nigra* | Reunion Is. | Pacific Island Ecosystems at Risk (PIER) | Not included | (Lavergne 2006) |
| *Pleioblastus fortunei* | United States | A Global Compendium of Weeds (Randall, 2012) | Not included | (Anon 2005) |
| *Phyllostachys flexuosa* | New Caledonia | Pacific Island Ecosystems at Risk (PIER) | Not included | (MacKee 1994) |
| *Bambusa vulgaris* | Australia | A Global Compendium of Weeds (Randall, 2012) | Not included | (Anon. 2009; Randall 2007) |
| *Phyllostachys bambusoides* | Australia | A Global Compendium of Weeds (Randall, 2012) | Not included | (Randall 2007; Anon 2009) |
| *Phyllostachys edulis* | Australia | Independent search | Not included | (Randall 2007) |
| *Phyllostachys nigra* | Australia | Pacific Island Ecosystems at Risk (PIER) | Not included | (Randall 2007; Csurhes & Edwards, 1998) |
| *Bambusa vulgaris* | Cook Is. | Pacific Island Ecosystems at Risk (PIER) | Not included | (Space & Flynn 2002) |
| *Phyllostachys bissetii* | Cook Is. | Pacific Island Ecosystems at Risk (PIER) | Not included | (Space & Flynn 2002) |
| *Bambusa vulgaris* | Costa Rica | A Global Compendium of Weeds (Randall, 2012) | Not included | (IABIN, 2008) |
| *Bambusa vulgaris* | Federated States of Micronesia | Pacific Island Ecosystems at Risk (PIER) | Not included | (Fosberg et al. 1987) |
| *Bambusa vulgaris* | Fiji | Pacific Island Ecosystems at Risk (PIER) | Not included | (Smith 1979) |
| *Bambusa vulgaris* | Guadeloupe | Independent search | Not included | (DEAL, 2011) |
| *Bambusa vulgaris* | Madagascar | A Global Compendium of Weeds (Randall, 2012) | Not included | (eFlora 2006) |
| *Bambusa vulgaris* | Martinique | Independent search | Not included | (DEAL, 2011) |
| *Bambusa vulgaris* | Mayotte Is. | Pacific Island Ecosystems at Risk (PIER) | Not included | (Comité français 2013) |
| *Bambusa vulgaris* | Mexico | A Global Compendium of Weeds (Randall, 2012) | Not included | (Conabio 2006) |
| *Phyllostachys aurea* | Mexico | A Global Compendium of Weeds (Randall, 2012) | Not included | (Conabio 2006) |
| *Bambusa balcooa* | Namibia | A Global Compendium of Weeds (Randall, 2012) | Not included | (Bethune et al. 2004) |
| *Bambusa vulgaris* | New Caledonia | Independent search | Not included | (Heque et al. 2009) |
| *Bambusa multiplex* | New Zealand | Pacific Island Ecosystems at Risk (PIER) | Not included | (Edgar & Connor, 2000) |
| *Bambusa vulgaris* | New Zealand | Invasive Species Compendium (CABI) | Not included | (Edgar & Connor, 2000) |
| *Phyllostachys nigra* | New Zealand | Pacific Island Ecosystems at Risk (PIER) | Not included | (Edgar & Connor, 2000) |
| *Bambusa vulgaris* | Niue | Invasive Species Compendium (CABI) | Not included | (Space et al. 2004) |
| *Bambusa vulgaris* | Tonga | Pacific Island Ecosystems at Risk (PIER) | Not included | (Space & Flynn 2001) |
| *Bambusa vulgaris* | United States | Independent search | Not included | (Swearingen et al. 2010) |
| *Pseudosasa japonica* | United States | Independent search | Not included | (Swearingen et al. 2010) |
| *Bambusa vulgaris* | Wallis-Futana Is. | Pacific Island Ecosystems at Risk (PIER) | Not included | (Morat & Veillon 1985) |

**References**

Anon. 2005. New England Bamboo Company Online Catalog. URL: www.newengbamboo.com/catalog/catalog.htm

Anon. 2009. Schedule 2 - Table 1. Amended List of Schedule 2 (Undesirable plants) in Undesirable Plants and Animals. Wet Tropics Management Authority, Cairns QLD. in Wet Tropics Plan

Review Wet Tropics Management Plan (1998). URL: http://www.wettropics.gov.au/mwha/mwha_planreview.html Bargeron, C.T., D.J. Moorhead, G.K. Douce, R.C. Reardon and A.E. Miller (Tech. Coordinators). 2003. Invasive Plants of the Eastern U.S.: Identification and Control. USDA Forest Service - Forest Health Technology.

Bethune S, Griffin M, Joubert D (2004) National Review of Invasive Alien Species Namibia. Consultancy Report on Information Collected Regarding Invasive Alien Species in Namibia for the Sabsp (Southern Africa Biodiversity Support Programme) Ministry of Environment and Tourism Directorate of Environmental Affairs, 153 pp.

Blundell AG. 2003. Ecorisk assessment using indicators of sustainability: invasive species in the Caribbean National Forest of Puerto Rico. Journal of Forestry 101:14.

Chacón E, Saborío G (2012) Red Interamericana de Información de Especies Invasoras, Costa Rica. San José, Costa Rica: Asociación para la Conservación y el Estudio de la Biodiversidad. URL: http://invasoras.acebio.org

Charles Darwin Foundation (2008) Database inventory of introduced plant species in the rural and urban zones of Galapagos. Galapagos, Ecuador: Charles Darwin Foundation.

Comité français de l'Union Internationale pour la Conservation de la Nature en France (2008) Espèces exotiques envahissantes dans les collectivités françaises d’outre-mer. URL: www.uicn.fr

Comité français de l'Union Internationale pour la Conservation de la Nature en France (2013) Les espéces envahissantes en outre-mer (online resource). URL: http://www.especesenvahissantes-outremer.fr/autoComplete/index.php

Conabio (2006) Programa de especies invasoras. Especies invasoras plantas (Mexico) URL: http://www.conabio.gob.mx/conocimiento/info_especies/especies_invasoras/doctos/plantas.html

Csurhes S, Edwards R (1998) Potential environmental weeds in Australia: Candidate species for preventative control. Canberra, Australia. Biodiversity Group, Environment Australia. 208 pp. URL: http://www.weeds.gov.au/publications/books/pubs/potential.pdf

Direction De L’Environnement, De L’Amenagement Et Du Logement –Guadeloupe Et Martinique (DEAL) (2011) Diagnostic sur l’invasion biologique Aux Antilles Françaises Stratégie de suivi et de prévention. Départements de la Guadeloupe et de la Martinique. Phase 1: Etat des lieux des connaissances, Version 3.

Edgar E, Connor H (2000) Flora of New Zealand, vol. V: Gramineae. Manaaki Whenua Press. eFlora. 2006. Naturalised Flora of Madagascar. eFlora Catalogue. URL: http://www.eFlora.org. Fosberg, F. R., Sachet, M-H and O., Royce. 1987. A geographical checklist of the Micronesian

monocotyledonae. Micronesica 20:1-126.

Fukushima K, Usui N, Ogawa R, Tokuchi N (2015) Impacts of moso bamboo (Phyllostachys pubescens) invasion on dry matter and carbon and nitrogen stocks in a broad-leaved secondary forest located in Kyoto, western Japan. Plant Species Biology, 30: 81–95. doi: 10.1111/1442-1984.12066

Gargominy O, Bouchet P, Pascal M, Jaffre T, Tourneu JC (1996) Conséquences des introductions d'espèces animales et végétales sur la biodiversité en Nouvelle-Calédonie. Rev. Ecol. (Terre Vie) 51:375-401.

Gucker CL (2009) Phyllostachys aurea. In: Fire Effects Information System, [Online]. U.S. Department of Agriculture, Forest Service, Rocky Mountain Research Station, Fire Sciences Laboratory (Producer). URL: http://www.fs.fed.us/database/feis/.

Héquet V, Le Corre M, Rigault F, Blanfort V (2009) Les espèces exotiques envahissantes de Nouvelle-Calédonie. Nouméa : IRD, 87 p. multigr. IABIN. 2008. Brazil: Red interamericana de Informacion sobre Biodiversidad. Red de Informacion sobre Especies Invasoras (I3N). URL: http://i3n.institutohorus.org.br/list_especies.asp

IABIN (2012) Invasives Information Network, i3N Brasil. Instituto

Hórus. Inter-American Biodiversity Information Network. URL: http://i3n.institutohorus.org.br/brasil/

Kairo M, Ali B, Cheesman O, Haysom K, Murphy S (2003) Invasive species threats in the Caribbean Region. Report to The Nature Conservancy, 134 pp. URL: http://tinyurl.com/awoxlorhttp://www.issg.org/database/species/reference_files/Kairo et al, 2003.pdf

Keighery G, Keighery B (2009) Calystegia silvatica comments. (pers.comm. by Randall 2012)

Kikodze D, Memiadze N, Kharazishvili D, Manvelidze Z, Mueller-Schaerer, H (2010) The Alien Flora of Georgia. Second Edition, Swiss National Science Foundation, Swiss Agency for Development and Cooperation and SCOPES (project number IB73A0-110830).

Lavergne, C (2006) List des especes exotiques envahissantes a La Reunion (List of invasive introduced species in Réunion). Unpublished manuscript.

MacKee HS (1994) Catalogue des plantes introduites et cultivées en Nouvelle-Calédonie. Muséum National d'Histoire Naturelle, Paris, 164 p.

Morat, P, Veillon JM (1985) Contribution à la connaissance de la végétation et de la flore de Wallis & Futuna. Bull. Mus. Natn. Hist. Nat., Paris, 4e sér., 7, section B. Adansonia 3 : 259-329.

Okuda S (1997) Wild plants of Japan. Shogakukan, Tokyo (in Jpn).

Oppenheimer H (2011) New Hawaiian plant records for 2009. In: Evenhuis, Neal L. and Eldredge, Lucius G., eds. Records of the Hawaii Biological Survey for 2009-2010. Part II: Plants. Bishop Museum Occasional Papers. 110:5-10.

Oviedo PR, Herrera OP, Caluff MG et al. (2012) National list of invasive and potentially invasive plants in the Republic of Cuba - 2011. (Lista nacional de especies de plantas invasoras y potencialmente invasoras en la República de Cuba - 2011.) Bissea: Boletín sobre Conservación de Plantas del Jardín Botánico Nacional de Cuba, 6(Special Issue 1): 22-96.

Queensland Herbarium (2002) Invasive Naturalised Plants in Southeast Queensland, alphabetical by genus. Modified from: Batianoff GN, Butler DW (2002) Assessment of Invasive naturalized plants in south-east Queensland. Appendix. Plant Protection Quarterly 17, 27-34. 11 pp.

Randall RP (2012) A Global Compendium of Weeds. 2nd Edition. Department of Agriculture and Food, Western Australia.

Richardson DM, Pyšek P, Carlton JT (2011) A compendium of essential concepts and terminology in invasion ecology. In: Fifty years of invasion ecology. The legacy of Charles Elton. Richardson DM (ed.) Wiley-Blackwell, Oxford. pp. 409 - 420.

Smith, AC (1979) Flora Vitiensis nova: a new flora of Fiji. National Tropical Botanical Garden, Lawai, Kauai, Hawaii. Volume 1. 494 pp.

Space JC, Flynn T (2002) Report to the Government of the Cook Islands on invasive plant species of environmental concern. USDA Forest Service, Honolulu.

Space JC, Flynn T (2001) Report to the Kingdom of Tonga on invasive plant species of environmental concern. USDA Forest Service, Honolulu.

Space JC, Waterhouse BM, Newfield M, Cate B (2004) Report to the Government of Niue and the United Nations Development Programme: Invasive plant species on Niue followingCyclone Heta. UNDP NIU/98/G31 - Niue Enabling Activity. 80 pp.

Staples GW, Imada CT, Herbst DR (2002) New Hawaiian plant records for 2000. In: Evenhuis, Neal L. and Eldredge, Lucius G., eds. Records of the Hawaii Biological Survey for 2000. Part 1: Articles. Bishop Museum Occasional Papers. 68:3-18.

Swearingen J, Slattery B, Reshetiloff K, Zwicker S (2010) In: Plant Invaders of Mid-Atlantic Natural Areas (4th ed). Washington, DC: National Park Service and U.S. Fish and Wildlife Service.

Toshiya Y (2004) Email to Aliens-L Listserver. URL: http://cain.ice.ucdavis.edu/cgi-bin/aliens-l.cgi

Tunison JT, D’Antonio CM, Loh RK (2000) Fire and invasive plants in Hawai’i Volcanoes National Park. In Proceedings of the invasive species workshop: the role of fire in the control and spread of invasive species. Fire conference pp. 122-131.

Wagner WL, Herbst DR, Sohmer SH (1999) Manual of the flowering plants of Hawaii. Revised edition. Bernice P. Bishop Museum special publication. University of Hawai‘i Press/Bishop Museum Press, Honolulu. 1919 pp. (two volumes).

Zenni RD, Ziller SR (2011) An overview of invasive plants in Brazil. Brazilian Journal of Botany, 34(3): 431-446.


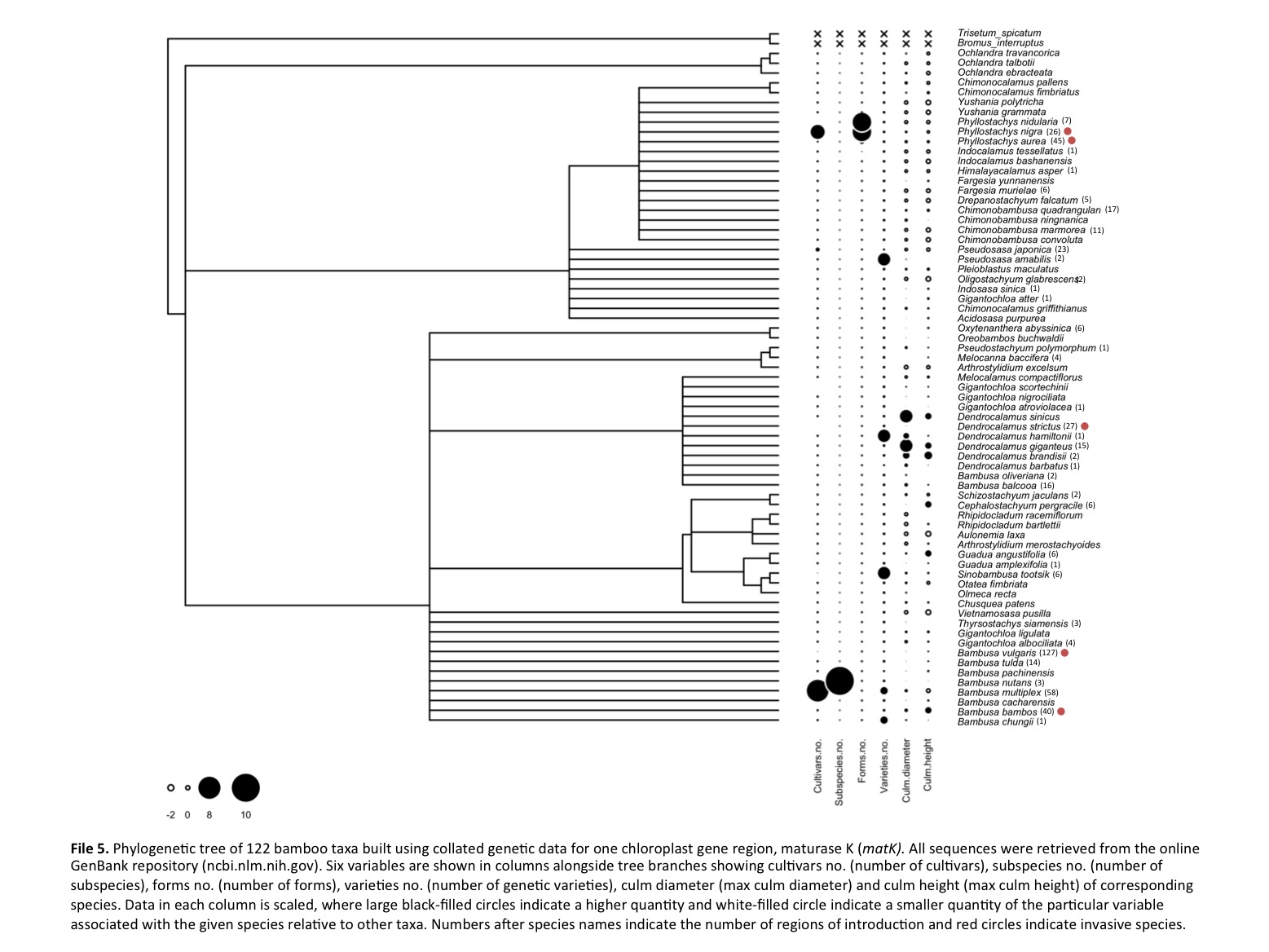


**Figure S2.** Phylogenetic tree of 122 bamboo taxa built using collated genetic data for one chloroplast gene region, maturase K (*matK).* All sequences were retrieved from the online GenBank repository (ncbi.nlm.nih.gov). Six variables are shown in columns alongside tree branches showing cultivars no. (number of cultivars), subspecies no. (number of subspecies), forms no. (number of forms), varieties no. (number of genetic varieties), culm diameter (max culm diameter) and culm height (max culm height) of corresponding species. Data in each column is scaled, where large black-filled circles indicate a higher quantity and white-filled circle indicate a smaller quantity of the particular variable associated with the given species relative to other taxa. Numbers after species names indicate the number of regions of introduction and red circles indicate invasive species.
